# Kinetic mechanism of controlled Fab-arm exchange for the formation of bispecific immunoglobulin G1 antibodies

**DOI:** 10.1074/jbc.RA117.000303

**Published:** 2017-11-17

**Authors:** Dennis R. Goulet, Steven J. Orcutt, Adam Zwolak, Theo Rispens, Aran F. Labrijn, Rob N. de Jong, William M. Atkins, Mark L. Chiu

**Affiliations:** From the ‡ Department of Medicinal Chemistry, University of Washington, Seattle, Washington 98195,; § Biologics Discovery, Janssen Research & Development, LLC, Spring House, Pennsylvania 19477,; the ¶ Sanquin Research and Landsteiner Laboratory, Department of Immunopathology, Academic Medical Centre, University of Amsterdam, Plesmanlaan 125, 1066 CX Amsterdam, The Netherlands, and; ‖ Genmab, Yalelaan 60, 3584 CM Utrecht, The Netherlands

**Keywords:** bispecific antibodies, antibody, cancer therapy, controlled Fab-arm exchange, fluorescence correlation spectroscopy (FCS), fluorescence resonance energy transfer (FRET), kinetics, reaction mechanism

## Abstract

Bispecific antibodies (bsAbs) combine the antigen specificities of two distinct Abs and demonstrate therapeutic promise based on novel mechanisms of action. Among the many platforms for creating bsAbs, controlled Fab-arm exchange (cFAE) has proven useful based on minimal changes to native Ab structure and the simplicity with which bsAbs can be formed from two parental Abs. Despite a published protocol for cFAE and its widespread use in the pharmaceutical industry, the reaction mechanism has not been determined. Knowledge of the mechanism could lead to improved yields of bsAb at faster rates as well as foster adoption of process control. In this work, a combination of Förster resonance energy transfer (FRET), nonreducing SDS-PAGE, and strategic mutation of the Ab hinge region was employed to identify and characterize the individual steps of cFAE. Fluorescence correlation spectroscopy (FCS) was used to determine the affinity of parental (homodimer) and bispecific (heterodimer) interactions within the C_H_3 domain, further clarifying the thermodynamic basis for bsAb formation. The result is a clear sequence of events with rate constants that vary with experimental conditions, where dissociation of the K409R parental Ab into half-Ab controls the rate of the reaction.

## Introduction

Bispecific antibodies (bsAbs)^2^ combine the antigen specificities of two parental Abs to create multifunctional molecules capable of simultaneously binding two distinct targets. In the pharmaceutical industry, bsAbs offer several biological mechanisms for treating disease that are not possible using traditional small molecule or monoclonal Ab therapies (1–4). For example, targeting two tumor antigens allows for inhibition of orthogonal signaling pathways and can increase specificity for cancer cells *versus* healthy tissue while also mitigating the incidence of resistance mechanisms (5–7). BsAbs can also facilitate co-localization of different cell types for applications such as T-cell redirection, where the immune system is activated against tumor cells (8). With two clinical approvals in 2009 and 2014 and many molecules in ongoing clinical trials, bsAbs are a relatively novel class of biotherapeutics whose development has been aided by protein engineering (1, 9, 10).

Currently, there are several platforms of bsAbs which can be categorized based on their inclusion of the Fc region (1, 11, 12). BsAb formats omitting the Fc domain, such as bispecific T-cell engagers (BiTEs) and dual-affinity retargeting proteins (DARTs), generally contain Ab variable fragments connected via polypeptide linkers (13, 14). Although the small size of these Abs relative to intact immunoglobulin G (IgG) may allow for increased penetration to solid tumors, the absence of the Fc region abrogates binding to Fc receptors responsible for mediating immune effector functions and long serum half-life (15, 16). Bispecific molecules based on the structure of full-length IgG retain Fc receptor binding but must be engineered to drive formation of the heavy chain heterodimer while retaining the correct pairing of heavy and light chains. The heavy chain pairing problem has been addressed by incorporating complementary mutations into the C_H_3 region using technologies such as controlled Fab-arm exchange (cFAE), knobs-into-holes, and electrostatic steering (17–19). cFAE uses a minimal set of mutations and avoids the light chain pairing issue, as exchange of half-Abs can be performed without perturbing the correct heavy chain–light chain interaction.

The cFAE reaction for formation of bispecific IgG1 was modeled after Fab-arm exchange (FAE) that occurs naturally in human IgG4, but not in other IgG subclasses (20–22). In general, the dimerization of heavy chains in the Fc relies on a combination of covalent interchain disulfide bonds mediated by hinge cysteine residues and noncovalent interactions primarily mediated by the C_H_3 domain. Like other isotypes, IgG4 Abs contain heavy chains that are normally linked by disulfide bonds in the hinge region. However, in IgG4, these hinge cysteines are contained within the motif “CPSC,” whereas other isotypes contain “CPPC.” The presence of serine at position 228 confers the hinge with extra flexibility that allows for the formation of intrachain disulfide bonds instead of the stabilizing interchain disulfide bonds (23). Additionally, IgG4 Abs contain arginine at position 409 (*versus* lysine in IgG1) which destabilizes the noncovalent interaction between heavy chains in the C_H_3 domain (24). This combination of a labile hinge region and a destabilized C_H_3 domain allows IgG4 to undergo half-Ab exchange (22). The F405L substitution similarly serves to decrease heavy chain dimerization affinity, but importantly, the interaction of complementary mutants is energetically preferred over the interaction of either homodimer (17). For the sake of clarity, exchange of any pair of half-Abs is here referred to as “Fab-arm exchange”; the term “controlled Fab-arm exchange” is reserved for the process of generating bsAbs from parental Abs containing F405L/K409R mutations.

To create bispecific IgG1 using cFAE, two parental Abs are separately expressed with the F405L or K409R mutations. Following purification, the parental Abs are combined under mild reducing conditions to selectively reduce the hinge disulfide bonds between parental heavy chains. Upon re-oxidation, intact bsAb is generated with yields over 90% (17, 25). The formation of therapeutic bsAbs is mechanistically more complex than the naturally occurring exchange that takes place with IgG4 molecules, because two heavy chains with chemically distinct C_H_3 domains allow for three possible half-Ab interactions. As a result, each parental Fc dissociation rate must be considered and could contribute to the overall rate of the reaction.

Although cFAE is used for the formation of several bispecific therapeutic IgGs in clinical trials, a full description of its mechanism has not been reported, and rates of its individual steps have not been accurately determined (26–28). Identification of rate-limiting steps of the reaction and conditions that affect the reaction rates will help to optimize large-scale production of bsAbs using cFAE and control the conjugation of drugs to the hinge cysteines of antibodies (29). A mechanistic analysis of cFAE is presented here, with description of each elementary step from oxidized parental Abs to oxidized bsAb product. The use of Förster resonance energy transfer (FRET) allowed for real-time monitoring of cFAE as half-Abs with donor or acceptor labels assembled into a single Ab molecule. Affinities for half-Ab homodimerization and heterodimerization were compared and confirmed a thermodynamic preference for bsAb over parental Abs. The rate constants of reduction and re-oxidation of inter–heavy chain disulfide bonds were determined based on nonreducing SDS-PAGE of the reaction contents over time. Together, the results elucidate a series of disulfide redox and protein-protein interaction events that can be experimentally regulated based on subtle changes to conditions such as pH and ionic strength.

## Results

### Rationale for hinge mutations

**Scheme 1.**
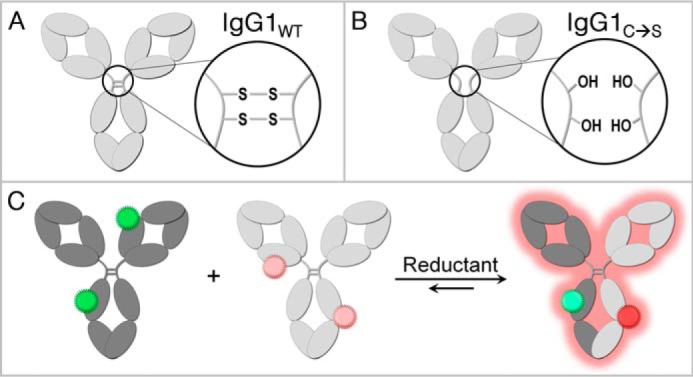
**Experimental tools for investigation of cFAE reaction mechanism.** *A*, human IgG1 with a wild-type hinge (IgG1_WT_) contains two disulfide bonds connecting half-Abs via the heavy chain. *B*, hinge mutants (IgG1_C→S_) contain serines in place of hinge cysteines, eliminating the covalent connection between half-Abs. *C*, the progress of cFAE is monitored using FRET, where each parental Ab is labeled with donor or acceptor fluorophore and fluorescence of the acceptor increases as bsAb is formed.

The formation of bsAb from two parental Abs requires the reduction of hinge disulfides, which allows for the exchange of half-Abs from parental homodimers to heterodimers. Parental IgG1 Abs with a native hinge contain a pair of inter–heavy chain disulfide bonds mediated by Cys-226 and Cys-229 from each half-Ab and therefore undergo exchange only under reducing conditions (IgG1_WT_, Scheme 1*A*). IgG1 hinge variants containing the C226S/C229S mutations provide a powerful mechanistic probe of cFAE (IgG1_C→S_, Scheme 1*B*). These mutations eliminate the covalent bonds between heavy chains and allow FAE to occur even in the absence of reductant. The IgG1_C→S_ constructs had typical human IgG1 expression levels and purification properties as well as normal bsAb yield via cFAE (Figs. S1–S3). Comparison of the kinetic profiles for cFAE with these constructs helped to elucidate which steps of the reaction involve redox chemistry at the hinge, and which are related to inter–heavy chain interactions occurring in the C_H_3 domain. Note that both IgG1_WT_ and IgG1_C→S_ Abs contain the appropriate cFAE mutations (F405L or K409R); the term wild-type for IgG1_WT_ refers to the Ab hinge region.

### Kinetics of cFAE

Progress of cFAE was monitored using FRET, with each parental Ab labeled with a different fluorophore (Scheme 1*C*). For example, an α-respiratory syncytial virus (RSV) Ab containing F405L was labeled with Alexa Fluor 488 as the FRET donor and an α-gp120 Ab containing K409R was labeled with Alexa Fluor 594 as the acceptor. The labeled Abs were then combined in equimolar amounts, and upon reduction of hinge disulfides, the Abs undergo FAE to form the bsAb which contains both fluorophores. In this bsAb, donor and acceptor dyes are within the Förster radius of ∼60 Å, and FRET increases the fluorescence of the acceptor fluorophore (30). The increase in FRET signal provides a real-time readout of bsAb formation and can be used to characterize the kinetics of cFAE (22, 31). When cFAE was performed with two distinct sets of IgG1 Abs containing cFAE mutations, there was no significant difference in the observed rates (Fig. S4). However, cFAE proceeded slightly faster for IgG1 Abs than the corresponding IgG4 S228P L234A L235A subtype Abs (Fig. S5).

### Effect of Ab concentration

**Figure 1.**
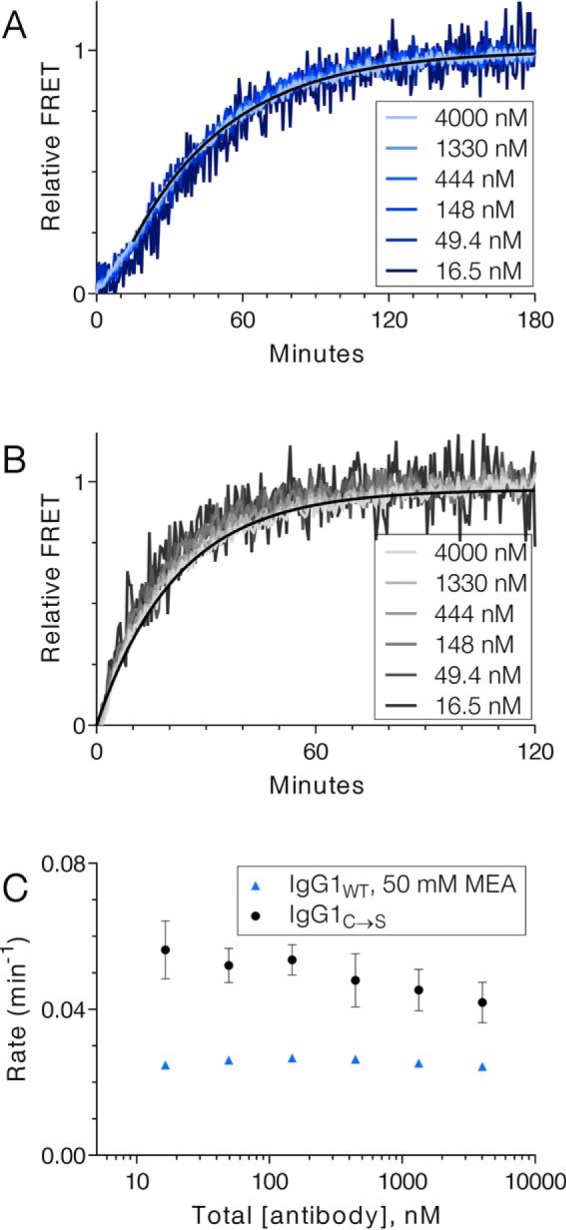
**Rate dependence of cFAE on Ab concentration.** *A* and *B*, cFAE progress curves for the formation of bispecific IgG1_WT_ (A) or IgG1_C→S_ (B) overlap when the reaction is performed with varying concentrations of Ab. The monoexponential fit is shown only for the 4 μm condition for the sake of clarity. *C*, the rate summary indicates the fitted rate of cFAE as a function of total Ab concentration.

To determine whether association of Abs or half-Abs is involved in the rate-determining step of cFAE, the reaction was performed with varying concentrations of parental Ab. If the slow step of cFAE were association of half-Abs to full Ab or association of intact Abs to Ab dimer, the reaction would proceed faster with increasing Ab concentrations. In contrast, if the rate-limiting step were a dissociation event, the overall rate of cFAE would be concentration independent. FRET was used to monitor the cFAE reaction using various concentrations of IgG1_WT_ or IgG1_C→S_ Abs. The rate was not affected by the concentration of antibody over the range of 16 nm to 4 μm, whether exchange was initiated by adding 2-mercaptoethylamine (MEA) to a mixture of parental IgG1_WT_ Abs or by combining parental IgG1_C→S_ Abs (Fig. 1). This result eliminates the possibility that association controls the overall rate, but instead indicates that dissociation to half-Ab is rate-limiting. For IgG1_WT_, (Fig. 1*A*) there was a lag phase in the kinetic trace for the first 15 min of the reaction. Because this lag is not seen for the IgG1_C→S_ constructs (Fig. 1*B*), it could be caused by the hinge reduction step. Thus, the requirement for an initial reduction of hinge disulfides reduces the rate of cFAE for IgG1_WT_ compared with IgG1_C→S_ and causes a lag phase at early extent of reaction (Fig. 1*C*).

### Effect of reducing agent

The role of the reducing agent was further explored by performing cFAE in the presence of different concentrations of MEA or dithiothreitol (DTT). For IgG1_WT_, there was a strong rate dependence on the concentration of MEA or DTT used to initiate the reaction (Fig. S6, *A* and *B*), with higher reductant concentrations leading to faster rates. In general, the rates with MEA were slower than those with an equivalent concentration of DTT, which is consistent with the stronger reduction potential of DTT (32, 33). The type and concentration of reducing agent played a less significant role when the reaction was performed with IgG1_C→S_. The concentration of MEA did not significantly affect the rate of cFAE for these hinge mutants (Fig. S6*C*), nor did the concentration of DTT alter the rate at concentrations below 25 mm (Fig. S6*D*). However, the reaction was accelerated in the presence of very high concentrations of DTT.

**Figure 2.**
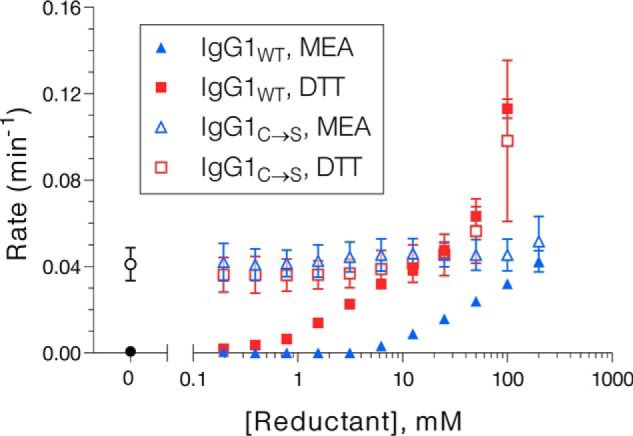
**Rates of cFAE for IgG1_WT_ and IgG1_C→S_ in the presence of various concentrations of MEA or DTT.** Rates with no reductant are plotted as a *closed black circle* (IgG1_WT_) or an *open black circle* (IgG1_C→S_). The reaction rate was faster than the plotted range in the presence of 200 mm DTT for both IgG1_WT_ (4.70 × 10^−3^ ± 0.28 × 10^−3^ s^−1^) and IgG1_C→S_ (4.85 × 10^−3^ ± 1.27 × 10^−3^ s^−1^). Rates shown are from monoexponential fits to kinetic FRET data shown in Fig. S3.

A summary of these experimental results (Fig. 2) illustrates that cFAE does not proceed in the absence of reducing agent for IgG1_WT_, but proceeds at an appreciable rate for IgG1_C→S_ even without reducing agent. As the concentration of MEA or DTT is increased, the rate of exchange remains constant for IgG1_C→S_, because hinge reduction is not necessary. The rate of cFAE for IgG1_WT_ increases from zero until reaching a plateau that is similar to the rate for IgG1_C→S_. In both cases, high concentrations of DTT accelerate the reaction beyond this plateau. Because cFAE occurs at similar rates under these conditions in the absence or presence of hinge disulfides, it is likely that the effects of DTT are mediated at other sites within the proteins.

### Effects of pH and ionic strength

**Figure 3.**
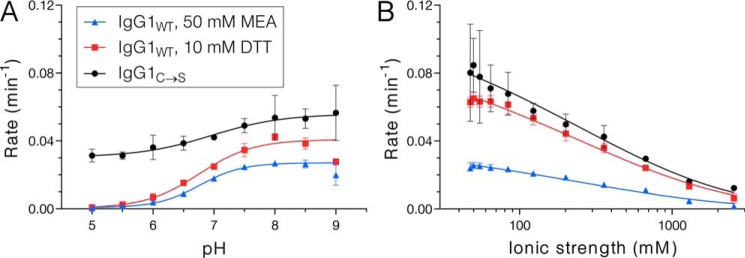
**Rates of cFAE at varying pH and ionic strength.** *A*, the effect of pH was investigated by performing the reaction in 100 mm potassium phosphate, 100 mm sodium chloride at pH 5 to 9. *B*, the effect of ionic strength was investigated by performing the reaction in 20 mm potassium phosphate, pH 7.4 containing 0 to 2.5 m sodium chloride. Rates shown are from monoexponential fits to kinetic FRET data shown in Figs. S4 and S5.

Because IgG1 hinge reduction clearly contributes to the rate of cFAE, and because disulfide chemistry involves thiolates as the active species (34), the reaction was expected to proceed faster at higher pH for IgG1_WT_. In fact, cFAE with IgG1_WT_ came to completion faster at higher pH in the presence of 50 mm MEA (Fig. S7*A*) or 10 mm DTT (Fig. S7*B*). Although the minimum rates were larger, there was also a pH dependence for IgG1_C→S_, which suggests that the pH effect is not entirely the result of thiol redox chemistry at the hinge (Fig. S7*C*). The effect of pH is more apparent when plotting the fitted rate of the reaction at each pH value (Fig. 3*A*) and emphasizes that the reaction is greatly hindered by lowering the pH to <6 for IgG1_WT_. For IgG1_C→S_ the effect is similar but less dramatic. The p*K_a_* values recovered from sigmoidal fits of the rate *versus* pH curves are similar for all conditions: 6.77 ± 0.04 for IgG1_WT_ with 50 mm MEA, 6.76 ± 0.08 for IgG1_WT_ with 10 mm DTT, and 6.96 ± 0.44 for IgG1_C→S_. When cFAE is performed with Abs containing a native hinge, the reaction appears to proceed fastest at pH 8, regardless of whether MEA or DTT is used.

Similarly, changes in ionic strength were expected to alter the reaction kinetics because of the dominance of charge effects at low ionic strength and of hydrophobic interactions at high ionic strength. Thus, cFAE was performed in buffers containing 0 to 2.5 m NaCl, which resulted in a similar trend in the rate profiles of IgG1_WT_ with 50 mm MEA (Fig. S8*A*), IgG1_WT_ with 10 mm DTT (Fig. S8*B*), and IgG1_C→S_ (Fig. S8*C*). Rate *versus* ionic strength curves could be described by a Debye-Hückel–like approximation that has been used previously (35, 36). Although the reaction with MEA reached a slower maximum rate, the reaction clearly proceeded more quickly in buffers with lower ionic strength (Fig. 3*B*). The maximum rate occurs when the ionic strength is <100 mm regardless of hinge mutations or type of reductant. This result presents two possibilities for the rate-limiting dissociation step. First, the half-Ab interface in the C_H_3 could contain repulsive electrostatic interactions that are accentuated in the low salt condition, leading to increased dissociation rate. Second, the half-Ab dimerization interface may contain stabilizing hydrophobic interactions which are dampened at low salt, again resulting in faster dissociation as salt is decreased. In either case, the similarity of trends for IgG1_WT_ and IgG1_C→S_ indicates that salt effects occur because of protein-protein interactions in the C_H_3 rather than redox chemistry at the hinge.

### Kinetics with different pairs of parental Abs

**Figure 4.**
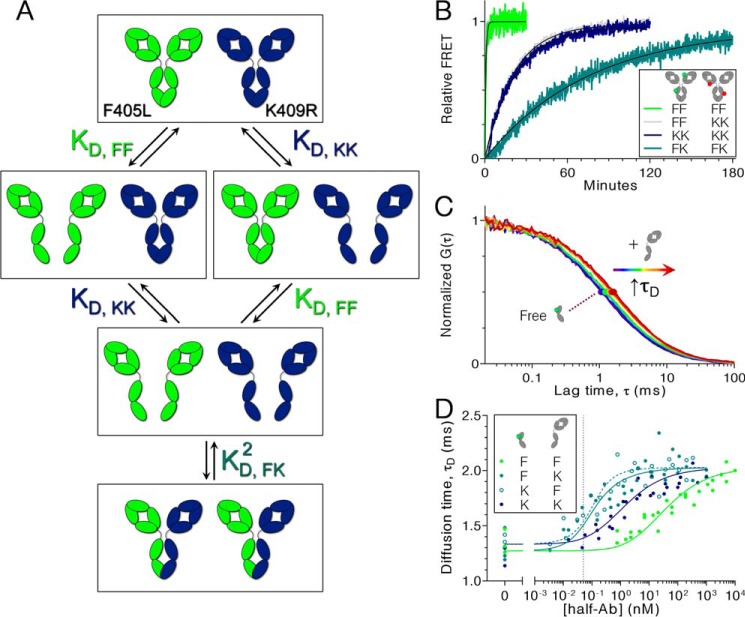
**Rate of FAE for different pairs of Abs reveals relative half-Ab dissociation rates, which correlate with half-Ab dimerization affinities.** *A*, the thermodynamics of bsAb formation involve discrete energetic states with combinations of associated and dissociated half-Ab. Each transition is defined by an equilibrium dissociation constant, *K_D_*, for either the F405L half-Ab homodimerization (*FF*), the K409R half-Ab homodimerization (*KK*), or the F405L/K409R half-Ab heterodimerization (*FK*). *B*, FAE progress curves for different combinations of labeled Abs show the slowest half-Ab dissociation rate involved in the exchange reaction. Parental F405L IgG1_C→S_ (*FF*), K409R IgG1_C→S_ (*KK*), or preformed IgG1_C→S_ bsAb (*FK*) were labeled with FRET donor or acceptor and FAE was monitored by FRET. *C* and *D*, FCS binding titrations were performed to determine *K_D_* values shown in *panel A. Panel C* shows one dataset for Alexa Fluor 488–labeled K409R half-Fc binding to unlabeled K409R half-Ab (IgG1_C→S_) for determination of *K_D_*,_KK_. The experiment was performed with a constant 50 pm of half-Fc and 1:3 serial dilutions of half-Ab (maximum 500 nm (*red*) decreasing to no half-Ab (*purple*)). *D*, upon binding, there is an increase in the apparent size of the labeled Fc, resulting in a larger diffusion time (*solid circles*). Diffusion times from all FCS experiments were globally fit to extract each of the three binding affinities (fitting described in supporting material). Each data point shows the average of 3–10 replicates; S.D. is omitted for clarity but was on average 9.54% of the measured value. *Dotted line* at 50 pm represents the fixed concentration of labeled half-Fc used in titrations.

Although the data demonstrate that the rate of cFAE is controlled by dissociation into half-Abs, the dissociation rate varies for each type of parental Ab because of the presence of the F405L or K409R mutations in the C_H_3 domain. Thus, the slower of these two dissociation events dictates the overall rate of the reaction. To determine the dissociation rates of each parental Ab into half-Ab, the FRET assay was used with different combinations of labeled parental IgG1_C→S_ (Fig. 4*B*). For example, the reaction with α-RSV F405L Alexa Fluor 488 and α-RSV F405L Alexa Fluor 594 reports on the rate of dissociation of the F405L parental Ab into half-Ab. In comparing the rate of each exchange reaction, the F405L parental Ab dissociates significantly faster than the K409R parental Ab (2.25 × 10^−2^ ± 0.62 × 10^−2^ *versus* 6.73 × 10^−4^ ± 0.22 × 10^−4^ s^−1^). Thus, the slower dissociation of the K409R parental Ab into half-Ab ultimately limits the rate of cFAE. Consistent with these observations, the rate of FAE for the K409R/K409R pair is like that of cFAE for the F405L/K409R pair (6.73 × 10^−4^ ± 0.22 × 10^−4^ and 7.72 × 10^−4^ ± 1.86 × 10^−4^ s^−1^) (Fig. 4*B*, *blue* and *gray curves*). Substituting the K409R parental with alternative Lys-409 mutations could increase the exchange rate (Fig. S9). When preformed bsAb was labeled with donor and acceptor fluorophores and allowed to exchange, the rate was the slowest (1.98 × 10^−4^ ± 0.05 × 10^−4^ s^−1^), indicating that dissociation of the half-Ab heterodimer is slower than dissociation of either homodimer.

### Equilibrium dissociation constants

Because binding affinity is proportional to dissociation rate (*K_D_* = *k_d_*/*k_a_*), the half-Ab dissociation rates measured by the kinetic assay were expected to correlate with the equilibrium dimerization affinities (Fig. 4*A*). To measure *K_D_* values for different pairs of Fc mutants, binding titrations were performed and the fraction bound at each concentration was determined by fluorescence correlation spectroscopy (FCS). This technique monitors the apparent size of a fluorescently labeled particle based on its rate of diffusion through the ∼1-femtoliter focal volume. To maximize the difference in size between bound and unbound states (and therefore maximize signal), titrations were performed with a fixed concentration of labeled half-Fc (∼25 kDa) in the presence of varying concentrations of unlabeled half-Ab (IgG_C→S_, ∼75 kDa). Upon binding of the labeled half-Fc to half-Ab, the ∼4-fold increase in mass leads to a change in translational diffusion time (τ_D_) that is observable by FCS. The sensitivity of FCS allowed very dilute concentrations to be explored (50 pm labeled half-Fc), which was crucial given the tight affinity of these interactions and ensured that most half-Fc was in the monomeric state in the absence of added half-Ab.

After obtaining FCS autocorrelations, curves were fit to a two-component three-dimensional diffusion model with one component fixed to the diffusion time of free dye. Because only ∼10% of fluorescence was from free dye, the two-component fits allowed for accurate determination of the τ_D_ of the protein component. Fig. 4*C* shows a representative dataset of FCS autocorrelations generated by titrating 50 pm Alexa Fluor 488–labeled K409R half-Fc with 0 to 500 pm K409R half-Ab. Resulting τ_D_ values were plotted against concentration of half-Ab to generate binding curves, which were fit to equations derived specifically for these FCS experiments (Fig. 4*D* and Scheme S3). The equations account for the competitive, non–two state nature of the experiment (because half-Fc and half-Ab may homodimerize or heterodimerize) by having *K_D_* values for each interaction. The *K_D_* values for F405L homodimerization, K409R homodimerization, and bsAb heterodimerization were determined by globally fitting datasets from all four half-Fc/half-Ab combinations. The resulting *K_D_* values were 12.2 ± 3.7, 0.337 ± 0.076, and 0.0656 ± 0.0343 nm for the F405L, K409R, and bsAb interactions, respectively, consistent with the trend for dissociation rates measured kinetically.

The *K_D_* values were also independently determined for an alternative parental antibody pair (α-EGFR IgG1 F405L and α-CD20 IgG1 K409R) (Fig. S10) using a different set of fluorophores and an SEC or FRET assay analagous to previous reports (31). Apparent dissociation constants were 191 ± 45, 2.8 ± 0.3, and 0.177 ± 0.010 nm for F405L, K409R, and bsAb, respectively, which for the K409R antibody is in close agreement to previous results (31). For the bsAb, the calculated value does not account for homodimerizations and is therefore only approximate. However, unlabeled bsAb was titrated to a fixed amount of fluorescently labeled F405L at concentrations where essentially no homodimerization took place, and any F405L-488 that eluted as a dimer therefore represented only heterodimers. Therefore, this experiment represents a conservative estimate of the *K_D_* value, which was overestimated to the extent that K409R could still form some homodimers and therefore negatively affects heterodimer formation. Again, the bsAb heterodimer interactions are substantially stronger than both the F405L and K409R homodimer interactions, explaining the preferential formation of bsAb over homodimers.

### Kinetics of hinge redox reactions

**Figure 5.**
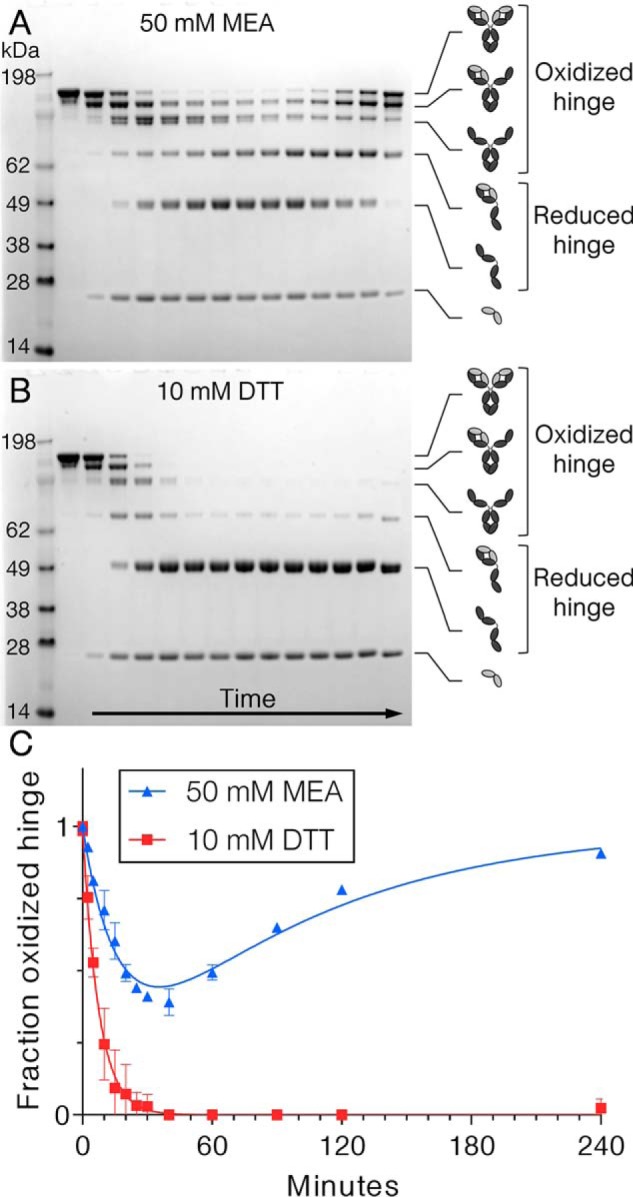
**Kinetics of hinge reduction and oxidation.** Nonreducing SDS-PAGE with iodoacetamide quenching was used to monitor the redox state of hinge disulfide bonds after 50 mm MEA (*A*) or 10 mm DTT (*B*) was added to initiate cFAE of IgG1_WT_. *C*, densitometry was then used to calculate the percentage of hinge disulfides that were oxidized at each time point using the densities of bands of oxidized heavy chains (150-, 125-, and 100-kDa) and reduced heavy chains (75- and 50-kDa). MEA data were fit to the sum of two exponentials to extract the fast rate of reduction and slower rate of re-oxidation. For DTT, the reduction rate alone was determined using a single exponential fit.

Because hinge reduction occurs more quickly than half-Ab exchange, FRET cannot be used to characterize the rates of Ab reduction and re-oxidation. Instead, nonreducing SDS-PAGE with iodoacetamide quenching was used for this purpose. At the start of the experiment, cFAE of IgG1_WT_ was initiated using 50 mm MEA or 10 mm DTT. Then at each time point, a reaction aliquot was removed and free thiols in the reaction were quenched with an excess of the alkylating agent to prevent further disulfide exchange from occurring. Samples were analyzed by nonreducing SDS-PAGE to monitor the change in Ab redox states with time (Fig. 5, *A* and *B*). Densitometry was used to calculate the percentage of Ab hinge that was oxidized at each time point, and the resulting kinetic trace was fit to a sum of two exponential equations to yield rates of reduction and re-oxidation (Fig. 5*C*). The rate of reduction of inter–heavy chain disulfide bonds was 9.34 × 10^−4^ ± 1.80 × 10^−4^ s^−1^ for 50 mm MEA and 2.23 × 10^−3^ ± 0.12 × 10^−3^ s^−1^ for 10 mm DTT. From the second exponential component, re-oxidation to form inter–heavy chain disulfides occurred at a rate of 1.80 × 10^−4^ ± 0.08 × 10^−4^ s^−1^ with 50 mm MEA; re-oxidation did not occur to a significant extent in 4 h in the presence of 10 mm DTT. To account for the possibility that rates of hinge redox reactions could be framework dependent, the procedure was performed for each parental Ab as well as the combination of Abs. However, the reduction profiles for α-RSV F405L, α-gp120 K409R, and the mixture were similar with rates of 1.48 × 10^−3^ ± 0.04 × 10^−3^, 1.49 × 10^−3^ ± 0.07 × 10^−3^, and 1.54 × 10^−3^ ± 0.06 × 10^−3^ s^−1^, respectively at 31 °C (Fig. S11). The re-oxidation kinetics were also similar, with rates of 4.60 × 10^−4^ ± 0.15 × 10^−4^, 4.00 × 10^−4^ ± 0.26 × 10^−4^, and 4.19 × 10^−4^ ± 0.24 × 10^−4^ s^−1^ for F405L parental, K409R parental, and the mixture to form bsAb.

### Mechanistic model of cFAE

**Figure 6.**
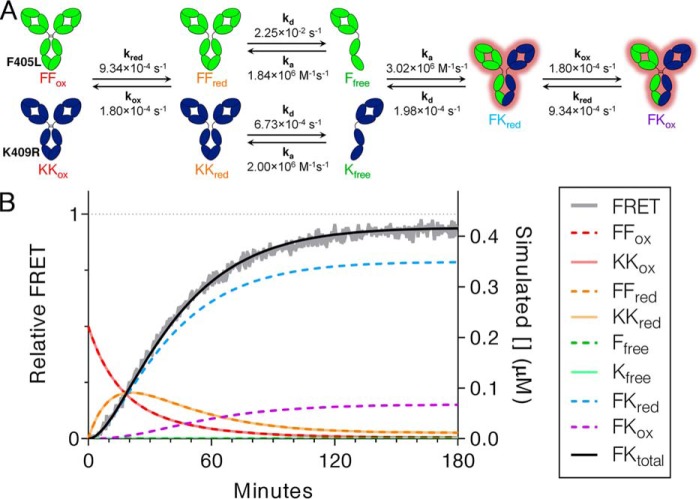
**Kinetic scheme for the mechanism of cFAE including measured rates of each step at 25 °C.** *A*, hinge disulfides of parental Abs must first be reduced (rate shown is with 50 mm MEA), followed by dissociation into half-Abs, re-association to form half-Ab heterodimer, and re-oxidation of hinge disulfides (again, rate shown is in the presence of 50 mm MEA) to stabilize the bispecific product. *B*, the kinetics of cFAE were simulated at an intermediate Ab concentration of 444 nm to determine whether this model agrees with FRET data under these conditions. The simulated concentration of each species is shown along with kinetic FRET data, which aligns well with the total concentration of bispecific Ab present. A *dotted line* at relative FRET = 1 and total [bsAb] = 444 nm indicates the maximum yield of bsAb if all parental Ab were converted to bsAb product.

These experiments yield a mechanistic model for the elementary steps of cFAE that occur on the path from oxidized parental Abs to oxidized bsAb for IgG1_WT_ (Fig. 6*A*). First, the inter–heavy chain disulfide bonds of parental Abs must be reduced, which occurs at a similar rate for the different Abs used here. The indicated rate was observed using 50 mm MEA as a reducing agent, but reduction kinetics will vary with the concentration and type of reductant as shown in Fig. 2. Next, the parental Abs with reduced hinge disulfides undergo dissociation into half-Abs, which occurs at different rates for the two cFAE mutants. Importantly, the much slower rate of dissociation of the K409R parental Ab is what ultimately determines the rate of cFAE. After dissociation occurs, half-Abs re-associate to form the intact bsAb containing reduced hinge disulfides. This half-Ab heterodimerization competes with homodimerization to parental Ab, but is driven toward the thermodynamically preferred heterodimer because of interactions at the C_H_3 domain. Finally, the bispecific product is re-oxidized to form disulfide-stabilized bsAb. The rate of re-oxidation shown is after reduction with 50 mm MEA but is expected to be faster if excess reductant is removed via dialysis.

This model was simulated and compared with FRET data from a cFAE reaction containing 222 nm each parental Ab and 50 mm MEA (Fig. 6*B*). The simulation provides the concentration of relevant species as cFAE proceeds. Strikingly, the simulated concentration of half-Ab heterodimer species accurately describes the experimental FRET data. The simulation also describes other relevant species that are more difficult to monitor experimentally, thus clarifying the maximum concentration and lifetime of intermediates. Although significant levels of reduced half-Ab homodimers build up early in the reaction, the free half-Abs exist only transiently at this concentration. The energetic instability of the half-Ab intermediates is consistent with half-Ab dissociation as the rate-determining step of cFAE; after half-Abs dissociate, they rapidly recombine to full Ab.

## Discussion

Although an understanding of the cFAE reaction is critically important for optimization of *in vitro* manufacturing, the precise chemical mechanism of bsAb formation using engineered IgG1 variants has not been established. Previously, a FRET assay was used to characterize the mechanism of FAE as it occurs naturally with human IgG4 (22). However, the kinetics of IgG1 cFAE presented here are substantially more complicated because of the exchange of two distinct half-Ab C_H_3 domains and the resulting combination of heavy chain interactions including two homodimerization equilibria and one heterodimerization equilibrium. Among the similarities, both mechanisms have parental Ab dissociation as the rate-determining step (22). Notably, Ab concentration did not affect the rate of cFAE, as there was no significant difference in rate at the bounds of concentrations used (16 nm to 4 μm) for either IgG1_WT_ (*p* = 0.173) or IgG1_C→S_ (*p* = 0.170). In the case of bsAb formation, it is the dissociation of the K409R parental Ab, rather than the F405L Ab, that ultimately controls the rate of cFAE. The rate profiles for IgG1_WT_ reduced with MEA or DTT are consistent with hinge reduction being a pre-equilibrium, rather than rate-limiting step, as was observed with IgG4 (22). Although higher concentrations of reductant resulted in faster reaction rates, the process was saturable. The IgG1_C→S_ mutants provide a clear upper limit for the rate of cFAE in the absence of hinge cysteines, and IgG1_WT_ reaches this limit only under strongly reducing conditions.

The hinge mutants additionally provided information on the role of interchain disulfides and C_H_3 interactions in each elementary step of cFAE. The C226S/C229S mutations have been used previously to prevent inter–heavy chain disulfide bond formation, but never in the context of the cFAE constructs or for mechanistic purposes (37, 38). By comparing the kinetics of cFAE for IgG1_WT_ and IgG1_C→S_, it was determined that ionic strength affects the dissociation of half-Abs based on C_H_3 interactions whereas pH affects thiol redox chemistry as well as protein-protein interactions at the C_H_3. The hinge mutants also facilitated the direct comparison of Ab dissociation rates and half-Ab *K_D_* values by removing the complexity associated with the hinge redox equilibrium.

SDS-PAGE experiments highlight the complexity of disulfide exchange at the hinge when MEA is used as the reducing agent. The fast hinge reduction was accompanied by an immediate, albeit slower, re-oxidation of free thiols. As a result, there was never a time when all hinge disulfides were in the reduced form. Kinetic FRET traces of bsAb formation for IgG1_WT_ showed a lag phase associated with hinge reduction; but even when fits omitted this phase, the reaction was slower than for IgG1_C→S_. An explanation for these SDS-PAGE and FRET data is that interchain disulfides at the hinge are in a dynamic equilibrium between the oxidized and reduced states. Even after sufficient exposure to MEA, only a population of interchain disulfides are reduced at any given time. The ability of MEA to form stable mixed disulfides with protein cysteines, rather than reducing them immediately to the free thiol, likely contributes to this complexity (39). Previously, hinge disulfides were shown not to undergo significant re-oxidation within the first 4 h of cFAE after reduction with 50 mm MEA (40). Although the cause for this discrepancy has not been determined, it could be because of differences in reaction volume (5 ml *versus* 0.1 ml), temperature (18–22 °C *versus* 25 °C), or other experimental variables.

Both IgG1_WT_ and IgG1_C→S_ underwent cFAE at an accelerated rate when DTT was present at concentrations above 25 mm, demonstrating that excess reducing agent can exhibit effects away from the hinge disulfides. This observation is not a result of light chain swapping because all interchain disulfide bonds of IgG1κ have been shown to have similar DTT reactivity (41), and the interaction between heavy chain and κ light chain is stable even when the interchain disulfides have been reduced and alkylated (42). Rather, the high concentrations of DTT probably allow for reduction of intrachain disulfide bonds, which causes an increased rate of half-Ab dissociation and thus of cFAE. It has been shown that the stability of immunoglobulin domains such as the C_H_3 are weakened upon intrachain disulfide reduction, and it is not unreasonable that this destabilization could increase the rate of half-Ab dissociation (43). Regardless of how this rate acceleration occurred, it did not affect other kinetic experiments performed under less-reducing conditions.

Determination of binding affinity constants was hindered by their extremely tight affinity and the complication that any mixture of the two cFAE mutants can form half-Ab homodimers as well as heterodimers. To address these considerations, experiments were performed at subnanomolar concentrations and fit to a model accounting for each possible species. FCS was a suitable technique for monitoring fraction bound, as it is functional under the dilute conditions that were required to reach the monomeric state. However, at such concentrations, FCS is truly a single molecule technique. The resulting data are poorly sampled, which allows individual replicates to sometimes vary widely from the true mean. Increased sampling was achieved by lengthening the collection time, increasing the number of replicates, and performing each titration on multiple days. Although the data still appeared noisy, there were clear differences in affinity that were clarified by globally fitting the datasets with shared parameters. Half-Ab dissociation rates correlated strongly to affinities (*R*^2^ = 1, Fig. S12), indicating that the association rate is quite similar for each interaction and that dissociation rate is the primary determinant of differences in affinity.

The *K_D_* values can also be used to calculate the equilibrium constant for the overall reaction and the theoretical yield of bsAb that would form for a fully reduced sample of cFAE mutants (see Schemes S1 and S2 for derivations). Using experimentally derived *K_D_* values, the expected yield of bsAb is 93.9%, which is consistent with values of >90% that are observed experimentally with methods such as hydrophobic interaction chromatography. Thus, there is a maximum achievable yield of bsAb defined by the relative affinities of parental and bispecific half-Ab dimerization. Although this percentage may vary with experimental conditions, the point remains that even perfectly executed cFAE reactions are subject to these thermodynamic limitations of yield. This sets an important benchmark for the process of cFAE.

It has been reported that the IgG4 Fc domain can interact with other molecules of intact IgG1 and IgG4 Fc (44, 45). Thus, the possibility of whole Ab heterodimerization, heavy chain exchange, and dissociation to bsAb was considered as an alternative to half-Ab dissociation and re-association. If the mechanism involved initial dimerization of intact Abs, then this complexation would be the step monitored by FRET and the observed kinetics would be dependent on Ab concentration. Because association occurs first by this mechanism, the FRET-observed reaction would be concentration dependent even if the rate-determining step occurred after association. The observed concentration independence of cFAE therefore precludes this Ab complexation mechanism and unambiguously identifies half-Ab dissociation as the slow step of the reaction.

Because cFAE is used to create large batches of bsAbs, the mechanistic details obtained here have implications for therapeutic bsAb production. It is clear from FRET that the IgG1_WT_ reaction with 50 mm MEA nears completion within 3 h at 25 °C, which is consistent with conditions cited in the protocol for large-scale cFAE incubations (25). Reactions to form bsAb are often performed in buffers at neutral pH, which is fortuitously in the pH range at which cFAE approaches its maximum rate. The data demonstrate that even a small increase in pH from 7.0 to 7.5 can result in a 32% increase in reaction rate because of a combination of accelerated hinge reduction and accelerated Ab dissociation. Likewise, decreasing the ionic strength of the reaction buffer from 200 to 80 mm results in a 28% increase in cFAE rate because of faster dissociation into half-Ab. Although high pH and low salt concentrations increase the rate of cFAE, there is no evidence to suggest that these conditions increase the final yield of bsAb. The reaction can also be accelerated using higher concentrations of reductant; however, the risk of intrachain disulfide reduction increases under extremely reducing conditions.

Comparison of experimental cFAE data and simulated data confirmed the validity of the mechanistic model and the accuracy of measured rates. The simulation itself incorporates data from three types of experiments to create a visual summary of cFAE that is both comprehensive and self-consistent. Furthermore, it highlights the remarkable efficiency of the cFAE reaction. Kinetically, a series of steps involving hinge reduction-oxidation and protein-protein interactions occurs at a rate determined by half-Ab dissociation and influenced by reduction conditions. Thermodynamically, the use of parental Abs with half-Ab dimerization affinities 5-fold to 180-fold weaker than that of the bispecific interaction ensures that cFAE will be driven toward the bsAb product. In the future, a similar experimental strategy could be used to characterize other processes involving multi-domain macromolecules.

## Experimental procedures

### Materials

Chemicals were purchased from Sigma. Abs against RSV (clone B21M, containing F405L) and HIV envelope glycoprotein gp120 (clone b12, containing K409R) in human IgG1 and human IgG1 lacking hinge disulfides (C226S/C229S) were expressed transiently in Expi293F cells (Thermo Fisher, A14527) according to the manufacturer's protocol (46, 47). Purification was performed using a HiTrap MabSelect SuRe column (GE Healthcare Life Sciences, 11–0034-94) according to the manufacturer's protocol, followed by immediate buffer exchange over a desalting column (GE Healthcare Life Sciences, 17–5087-01) into fresh phosphate-buffered saline (2.67 mm KCl, 1.47 mm KH_2_PO_4_, 138 mm NaCl, 8.06 mm Na_2_HPO_4_, pH 7.2). SDS-PAGE and analytical size-exclusion chromatography (SEC) were used to analyze proteins. Hydrophobic interaction chromatography was used to ensure that cFAE went to completion (>90% bsAb) for both IgG1_WT_ and IgG1_C→S_.

### Fluorescent labeling

Abs were diluted to 5 mg/ml in PBS containing 100 mm sodium bicarbonate, pH 8.3, and labeling was performed by adding a 6-fold molar equivalent of Alexa Fluor 488 or Alexa Fluor 594 NHS esters (Thermo Fisher, A-20000, A-20004) from a 10 mg/ml stock in DMSO. The reaction was incubated in the dark for 1 h at room temperature, followed by two rounds of buffer exchange (GE Healthcare Life Sciences, 28–9180-04). Concentration of labeled Ab and dye was calculated using absorbance as seen in Equations 1 and 2:

(Eq. 1) $$\left[\text{Ab}\right]=\frac{A_{\text{Ab}}-\left(A_{\text{dye}}\times \text{CF}\right)}{\epsilon_{\text{Ab}}}$$

(Eq. 2) $$\left[\text{Dye}\right]=\frac{A_{\text{dye}}}{\epsilon_{\text{dye}}}$$

where α-RSV F405L had ϵ_280_ = 220,000 M^−1^cm^−1^, α-gp120 K409R had ϵ_280_ = 231,000 M^−1^cm^−1^, the donor (Alexa Fluor 488) had ϵ_494_ = 73,000 M^−1^cm^−1^ and correction factor (CF) = 0.143 in PBS, and the acceptor (Alexa Fluor 594) had ϵ_590_ = 92,000 M^−1^cm^−1^ and CF = 0.564 in PBS. The final dye:Ab ratio was 1–2.

### Generation and purification of labeled Fc

After labeling 1 mg each of α-RSV F405L and α-gp120 K409R in IgG1_WT_ with 10× Alexa Fluor 488 as described above, Fc and F(ab′)_2_ were generated using IdeS enzyme (FabRICATOR, Genovis) according to the manufacturer's instructions. The Fc fraction was purified using protein A chromatography as described above. SEC was also performed using a Superdex 200 10/300 GL column (GE Healthcare Life Sciences, 17517501) in PBS at 0.5 ml/min to remove undigested or singly digested IgG and residual free dye. Purity of Fc domains was analyzed by SDS-PAGE (Fig. S13). The final dye:Ab ratio was ∼2.

### Kinetics of cFAE measured by FRET

Experiments were performed in black half-area 96-well plates (Corning, 3993) using 100 nm for each parental Ab-dye conjugate in 50 μl of PBS at 25 °C. The FAE reaction was initiated either by adding 50 mm MEA or 10 mm DTT to the mixture of IgG1_WT_ or by combining pairs of Abs in the case of IgG1_C→S_ and was monitored every 30 s (or every 10 s in the case of the different Ab pairs) by exciting at 494 nm and measuring emission at 617 nm (corresponding to fluorescence of the acceptor fluorophore) on a SpectraMax M5 plate reader. FRET signal was normalized by subtracting the minimum fluorescence and dividing by the maximum FRET in a dataset. cFAE reactions were performed once with the α-RSV F405L Alexa Fluor 488/α-gp120 K409R Alexa Fluor 594 pairing and once with the α-RSV F405L Alexa Fluor 594/α-gp120 K409R Alexa Fluor 488 pairing, with reported rates as the average ± S.D. The effect on kinetics of labeling with different amounts of dye was determined over a dye:Ab range of ∼0.5 to ∼15 (Fig. S14). Rates were determined using monoexponential fits in GraphPad Prism 7.

To clarify the effect of different Ab concentrations, the reaction was performed using 1:3 dilutions of Ab starting with 4 μm total Ab. To clarify the role of the reductant, MEA or DTT was added from 1:2 dilutions starting with a final concentration of 200 mm. To determine pH dependence, the reaction buffer was 100 mm potassium phosphate, 100 mm sodium chloride with a pH ranging from 5 to 9 in 0.5-pH increments. To determine salt dependence, the reaction buffer was 20 mm potassium phosphate containing 1:2 serial dilutions of sodium chloride, starting at 2.5 m. When comparing rates of FAE for different pairs of Abs, the concentration was increased to 200 nm for each parental Ab to increase signal, because only half of products were FRET active.

### Equilibrium dissociation constants measured by FCS

Experiments were performed on an Axio Observer D1 microscope (Zeiss) equipped with an LDH-D-C-485 picosecond pulsed diode 485 nm laser, Tau-SPAD single photon counting module, and HydraHarp 400 for autocorrelation (PicoQuant). Time traces and autocorrelations were collected using SymPhoTime 64 software (PicoQuant).

All buffers were filtered through a 0.22-μm filter prior to sample preparation. Samples contained a constant 50 pm of Alexa Fluor 488-labeled half-Fc and 1:3 or 1:4 dilutions of unlabeled half-Ab (IgG1_C→S_) and were equilibrated at room temperature overnight before analysis. Sample aliquots of 50 μl were placed onto 22 × 22–1 cover glass. With the laser power at 250 microwatts, several replicate traces were obtained for each sample and contained 200 sampling points and a maximum lag time of 10 s. Data collection intervals varied from 60 to 180 s in different datasets, but did not significantly affect measured diffusion times. Inclusion of two components significantly improved fits, where one component was fixed at the diffusion time of free Alexa Fluor 488 to account for residual free dye in samples. Traces were fit individually using Igor Pro over the range of 0.01 to 1000 ms using the two-component three-dimensional diffusion model in Equation 3 (48):

(Eq. 3) $$G\left(\tau \right)=\frac{1}{N}\left[\rho_{\text{dye}}\left(\frac{1}{1+\frac{\tau }{\tau_{D,\text{dye}}}}\right){\left(\frac{1}{1+\frac{\tau }{s^{2}\tau_{D,\text{dye}}}}\right)}^{0.5}+\rho_{\text{prot}}\left(\frac{1}{1+\frac{\tau }{\tau_{D,\text{prot}}}}\right){\left(\frac{1}{1+\frac{\tau }{s^{2}\tau_{D,\text{prot}}}}\right)}^{0.5}\right]$$

where *G*(τ) is the autocorrelation at a given lag time τ, *N* is the average number of particles in the focal volume, τ*_D,dye_* and τ*_D,prot_* are the translational diffusion times of the free dye and protein components, ρ*_dye_* and ρ*_prot_* represent the fraction of each component, and *s* is the ratio of long to short axes of the focal volume. The values of *s* and τ*_D,dye_* were fixed at 10 and 0.25 ms for all samples based on their values for free Alexa Fluor 488. The τ*_D_* of the protein component was the value used for binding curves. For all samples, the labeled half-Fc contributed >80% of the total signal with the remainder from the free dye component.

Binding curves plot the diffusion times from FCS *versus* the concentration of unlabeled half-Ab. Each point represents the average of 3–10 replicates, and each interaction contained datasets from 3 different days. Curves were globally fit to a custom fitting equation (Scheme S3) that accounts for half-Fc and half-Ab homodimerization in addition to the heterodimerization interaction.

### Disulfide redox kinetics

To determine rates of hinge reduction and re-oxidation, 1 mg/ml each of α-RSV F405L and α-gp120 K409R were reduced with 50 mm MEA or 10 mm DTT and incubated for 4 h at 25 °C with shaking at 300 rpm. At each time point, a 5-μl aliquot was removed from the reaction and quenched with 5 μl of 375 mm iodoacetamide (Thermo Fisher, 90034) in 200-mm sodium bicarbonate, pH 8.0. Later, all the quenched samples were analyzed by nonreducing SDS-PAGE on a NuPAGE 4–12% gels (Thermo Fisher, NP0323BOX), loading 3 μl per lane. Band densities were quantified using Image Lab software, and the percentage of oxidized hinge was calculated by dividing the sum of densities of the 150-, 125-, and 100-kDa bands by the sum of densities of the 150-, 125-, 100-, 75-, and 50-kDa bands. The datasets were fit to a sum of an exponential decay and an exponential association equation to extract reduction and re-oxidation rates. Each reaction was performed in duplicate with rates reported as average ± S.D.

### Simulation

The SimBiology toolbox of Mathematica R2017a was used to model cFAE kinetics. Equilibria included F405L half-Ab homodimerization, K409R half-Ab homodimerization, and half-Ab heterodimerization. The FAE rates measured by kinetic FRET experiments of IgG1_C→S_ antibodies were used for the *k_d_* of each binding reaction. The *k^a^* values were determined using *k_d_* values from FRET and *K_D_* values from FCS (*k^a^* = *k_d_*/*K_D_*). In addition to half-Ab binding, the equilibria between oxidized and reduced hinges were also included. The *k*_red_ and *k*_ox_ values were based on SDS-PAGE experiments with 50 mm MEA. The simulated reaction initially contained 222 nm of each oxidized parental Ab and was monitored of 180 min using the ode23t solver. The total amount of bsAb was defined as the sum of oxidized and reduced bsAb.

## Author contributions

D. R. G., S. J. O., T. R., R. N. d. J., W. M. A., and M. L. C. conceptualization; D. R. G., S. J. O., A. Z., T. R., A. L., and R. N. d. J. data curation; D. R. G. software; D. R. G., A. Z., T. R., A. L., W. M. A., and M. L. C. formal analysis; D. R. G., S. J. O., A. Z., T. R., A. L., R. N. d. J., W. M. A., and M. L. C. investigation; D. R. G. and T. R. visualization; D. R. G., S. J. O., A. Z., T. R., A. L., and R. N. d. J. methodology; D. R. G. writing-original draft; D. R. G., S. J. O., A. Z., T. R., A. L., R. N. d. J., W. M. A., and M. L. C. writing-review and editing; A. Z., A. L., R. N. d. J., W. M. A., and M. L. C. resources; A. L., W. M. A., and M. L. C. validation; W. M. A. and M. L. C. supervision; W. M. A. and M. L. C. funding acquisition; W. M. A. and M. L. C. project administration.

## Supplementary Material

Supporting Information

## Footnotes

2 The abbreviations used are:

bsAb: bispecific antibody
Ab: antibody
cFAE: controlled Fab-arm exchange
CF: correction factor
FCS: fluorescence correlation spectroscopy
gp120: glycoprotein 120
MEA: 2-mercaptoethylamine
PBS: phosphate-buffered saline
RSV: respiratory syncytial virus
SEC: size-exclusion chromatography.
