# Supplementary material for: Kinetic mechanism of controlled Fab-arm exchange for the formation of bispecific immunoglobulin G1 antibodies
Source: J Biol Chem. 2017 Nov 17;293(2):651–61. doi: 10.1074/jbc.RA117.000303 (PMC5767869; doi:10.1074/jbc.RA117.000303)
Supplement: Supporting Information [file supp_293_2_651__index.html]

Kinetic Mechanism of Controlled Fab-Arm Exchange for the Formation of Bispecific Immunoglobulin G1 Antibodies — Mechanism of controlled Fab arm exchange to make BsAbs — Kinetic mechanism of controlled Fab-arm exchange for the formation of bispecific immunoglobulin G1 antibodies — Mechanism of controlled Fab-arm exchange — Supporting Information 

# Kinetic mechanism of controlled Fab-arm exchange for the formation of bispecific immunoglobulin G1 antibodies

## Supporting Information

- Kinetic Mechanism of Controlled Fab-Arm Exchange for the Formation of Bispecific Immunoglobulin G1 Antibodies - Supplementary figures and tables
