# Supplementary material for: Kinetic mechanism of controlled Fab-arm exchange for the formation of bispecific immunoglobulin G1 antibodies
Source: J Biol Chem. 2017 Nov 17;293(2):651–61. doi: 10.1074/jbc.RA117.000303 (PMC5767869; doi:10.1074/jbc.RA117.000303)
Supplement: Supporting Information [file supp_RA117.000303_132943_0_supp_10670_wxdr51.pdf]

Supporting Information for:

Kinetic Mechanism of Controlled Fab-Arm Exchange for the Formation of Bispecific Immunoglobulin G1 Antibodies[*]

Dennis R. Goulet[‡], Steven J. Orcutt[§], Adam Zwolak[§], Theo Rispens[¶], Aran F. Labrijn[‖], Rob N. de Jong[‖], William M. Atkins[‡], and Mark L. Chiu[§1]

From the [‡]Department of Medicinal Chemistry, University of Washington, Seattle, Washington 98195; [§]Biologics Discovery, Janssen Research & Development, LLC, Spring House, Pennsylvania 19477; [¶]Sanquin Research and Landsteiner Laboratory, Department of Immunopathology, Academic Medical Centre, University of Amsterdam, The Netherlands; and [‖]Genmab, Utrecht, The Netherlands

[*]This work was supported, in whole or in part, by National Institutes of Health Grant T32GM007750 (to D.R.G.) and the University of Washington Department of Medicinal Chemistry. The content is solely the responsibility of the authors and does not necessarily represent the official views of the National Institutes of Health.
[1]To whom correspondence should be addressed: Janssen Research and Development, LLC, 200 Great Valley Parkway, Malvern, PA 19355. Tel.: 610-651-6862; E-mail: MChiu@its.jnj.com.

Contents:
S-2,3: Supplementary materials and methods
S-4: Figure S1 showing SDS-PAGE of $IgG1_{WT}$ and $IgG1_{C\rightarrow S}$ Abs
S-5: Figure S2 showing SEC of $IgG1_{WT}$ and $IgG1_{C\rightarrow S}$ Abs
S-6: Figure S3 showing HIC after cFAE reaction using $IgG1_{WT}$ and $IgG1_{C\rightarrow S}$ Abs
S-7: Figure S4 showing cFAE kinetics with two different pairs of $IgG1_{WT}$ Abs
S-8: Figure S5 showing cFAE kinetics for IgG1 and IgG4 Abs
S-9: Figure S6 showing raw data for cFAE under different reducing conditions
S-10: Figure S7 showing raw data for cFAE at different pH
S-11: Figure S8 showing raw data for cFAE at different ionic strength
S-12: Figure S9 showing cFAE for different K409 mutants
S-13: Figure S10 showing alternative measurement of half-Ab K_D values
S-14: Figure S11 showing the kinetics of hinge redox for parental and bsAbs
S-15: Figure S12 showing comparison of half-Ab k_d and K_D
S-16: Figure S13 showing SDS-PAGE of purified IgG1 Fc
S-17: Figure S14 showing the effect of dye:Ab ratio on cFAE kinetics
S-18: Scheme S1 showing derivation of K_{eq} for cFAE
S-19: Scheme S2 showing calculation of %bsAb based on half-Ab K_D values
S-20,21: Scheme S3 showing derivation of FCS fitting equations
S-22: Supplementary references

Supplementary Materials and Methods

Antibodies

Parental antibodies α-EGFR IgG1 F405L and α-CD20 IgG1 K409R, based on (HuMab) clones 7D8 (1) and 2F8 (2) respectively, and bsIgG1-EGFR-F405LxCD20-K409R were produced as described previously (3). Alternatively, the α-CD20 IgG1-K409X parental antibodies with alternative K409 substitutions were produced as described earlier (4). All parental and bispecific antibodies were labeled with DyLight-488 or DyLight-594 NHS esters (Thermo Fischer cat. No. 46402 and 46412) according to the manufacturer's instructions.
Parental antibodies α-TNFα (CNTO 148) IgG1 F405L and α-ITGAV (CNTO 95) IgG1 K409R were produced analogously to α-RSV and α-gp120 in the main text and labelled with Alexa 488 or Alexa 594 as described in the main text (5,6).

Kinetics of cFAE measured by FRET.

To determine the kinetics of parental pairs containing the α-CD20 IgG1 parental with alternative K409 substitutions, parental Ab-dye conjugates were mixed in a 1:1 ratio at a final concentration of 167 nM in 18 µL of PBS at 25 °C in 96-well PCR plates. The FAE reaction was initiated by adding 9 µL of 2-MEA (final concentration 25 mM) and incubating in an iQ™5 Multicolor Real Time PCR Detection System (BioRad) set at 25 °C. FAE was monitored every 2 minutes by exciting at 494 nm and measuring emission at 620 nm. FRET signal was normalized by subtracting the minimum fluorescence and dividing by the maximum FRET in a dataset. Rates were determined using Sigmoidal dose-response (variable slope) fits in GraphPad Prism 7.

Equilibrium constants (Fluorescence-assisted high-performance liquid chromatography)

To determine equilibrium constants, antibodies and DyLight-488 labeled antibodies were reduced with 10 mM DTT (60 min/37 °C) and alkylated with 22 mM of iodoacetamide. Serial four-fold dilutions of reduced/alkylated IgG (0.003 – 1000 nM half-molecules) were incubated with 0.1 ng/ml reduced/alkylated F405L-488 (for the bsAb) or 0.5 ng/ml K409R-488 (for the K409R antibody) in PBS containing as a carrier protein 0.1 mg/ml certolizumab pegol, and incubated at 37 °C for up to 1 day before analysis. Between 50 – 1000 µl of a sample was applied using a thermostatted autosampler (20 °C) to a Superdex 200 HR 10/300 column, which was connected to an ÄKTAexplorer HPLC, and eluted at 0.5 ml/min. Elution profiles were monitored by measuring the fluorescence (excitation/emission 488/525 nm) with a Prominence RF-20Axs in-line fluorescence detector (Shimadzu, Kyoto, Japan). To calculate dissociation constants, background-corrected fluorescence intensities (F) corresponding to the peak maxima of either bound or free labeled protein were plotted against the concentration of antibody x (molar concentration of the number of half-molecules), and a homodimerization model ($F = F_0 + 0.25 \times \Delta F \times [K_D + 4x - (K_D 2 + 8x \times K_D)^{0.5}]/x$), where F_0 is the fluorescence at zero concentration, ΔF is the fluorescence at saturating concentrations minus F_0, and K_D is the dissociation constant) was fitted to the data using Microcal Origin 7.0 software. For the bsAb, an apparent dissociation constant was measured as $F = F_0 + \Delta F \times [(x + y + K_D) - ((x + y + K_D)^2 - 4xy)^{0.5}]/2y$, with $x = y$ (molar concentrations of half-molecules of the K409R and F405L variants, resp). Standard errors were calculated from duplicate or triplicate values of the K_D as determined in independent experiments.

Equilibrium constants (FRET)

Equilibrium constants of F405L was determined by recording fluorescence spectra using a Nanodrop ND3300 of two-fold dilutions of equimolar mixtures of F405L -488 and F405L -594 after incubation for 2 hours at 37 ^{o}C in the presence of 3 mM DTT and subsequent equilibration at 20 ^{o}C. Spectra were corrected for background fluorescence and the ratio of F_{620}/F_{588} was plotted against total concentrations of half-molecules (A+B) to obtain a dose-response curve. The concentration of mixed F405L dimers (AB) will correlate linearly with the total amount of F405L dimers (A_2 + B_2 + AB), therefore, the FRET signal is representative of the amount of dimers present in solution. The dissociation constant was calculated by fitting a homodimerization model to the data (see above), reported value is the average of 5 different experiments.

Hydrophobic interaction chromatography (HIC)

HIC was used as an orthogonal technique to FRET to ensure formation of >90% bsAb for $IgG1_{WT}$ and $IgG1_{C \rightarrow S}$ Abs containing F405L/K409R mutations. After performing cFAE reactions including dialysis to remove reducing agent, 20 µg of protein was injected onto a butyl-NPR column (Tosoh Bioscience, 42168) and parental Abs were separated from bsAb using a gradient from 1.5 M to 0 M $(NH_4)_2SO_4$ in 0.1 M sodium phosphate, pH 6.5 at 0.5 mL/min. HIC of purified parental Abs was also performed to identify which protein each peak represents.

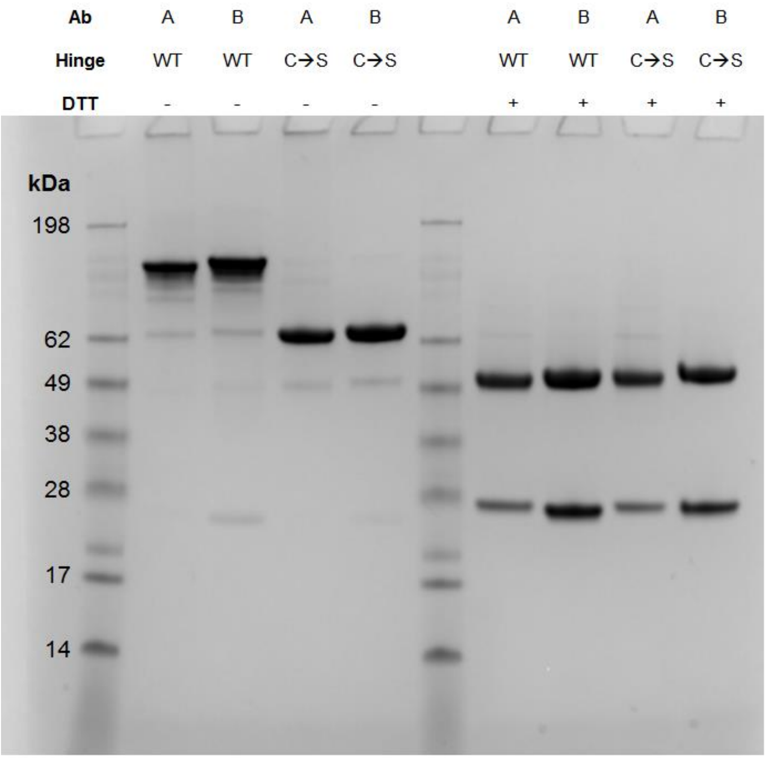

Figure S1. SDS-PAGE of Abs that were used to characterize cFAE kinetics. Ab A is α-RSV F405L and Ab B is α-gp120 K409R, both in huIgG1. Abs contained either a wild-type hinge region (WT), or contained C226S/C229S (C→S). Under non-reducing conditions (no DTT), Abs with a native hinge traveled as the expected 150 kDa species, while those containing hinge mutations appeared as a 75-kDa band indicative of half-Ab. When 50 mM DTT was added to samples, they were all shown to contain a 50-kDa heavy chain and a 25-kDa light chain. Experiment was performed by running a NuPAGE 4-12% gel (Thermo Fisher, NP0321BOX) at 150 V and loading 3 μg of protein per lane.

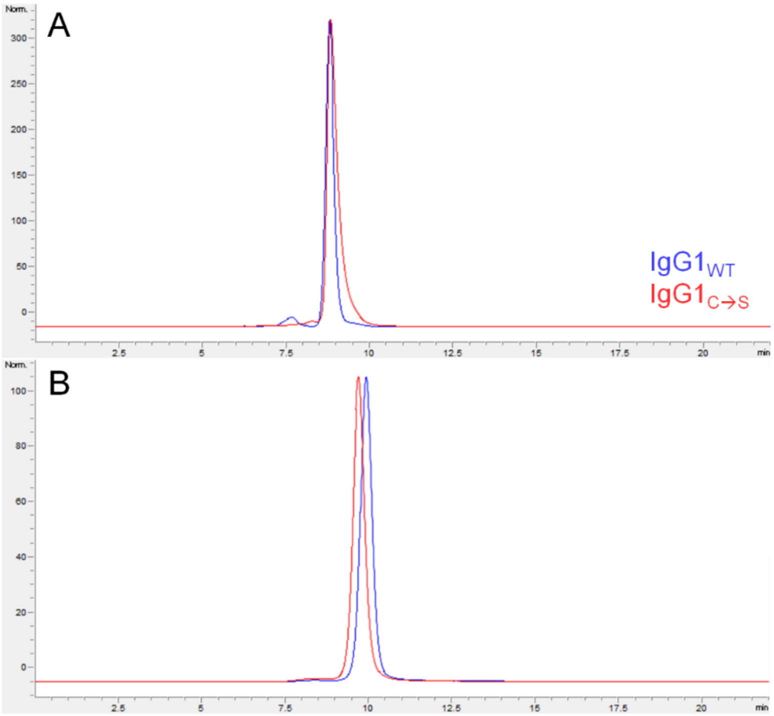

Figure S2. Size-exclusion chromatograms of α-RSV F405L (A) and α-gp120 K409R (B) demonstrate that wild-type ($IgG1_{WT}$) and C226S/C229S ($IgG1_{C\rightarrow S}$) behave similarly, with a single major peak at a retention time consistent with monomer. Twenty µg of each sample was run over a G3000SWxl column (Tosoh Bioscience, 08541) equilibrated in PBS at 1.0 mL/min, with plots showing relative absorbance at 280 nm versus retention time.

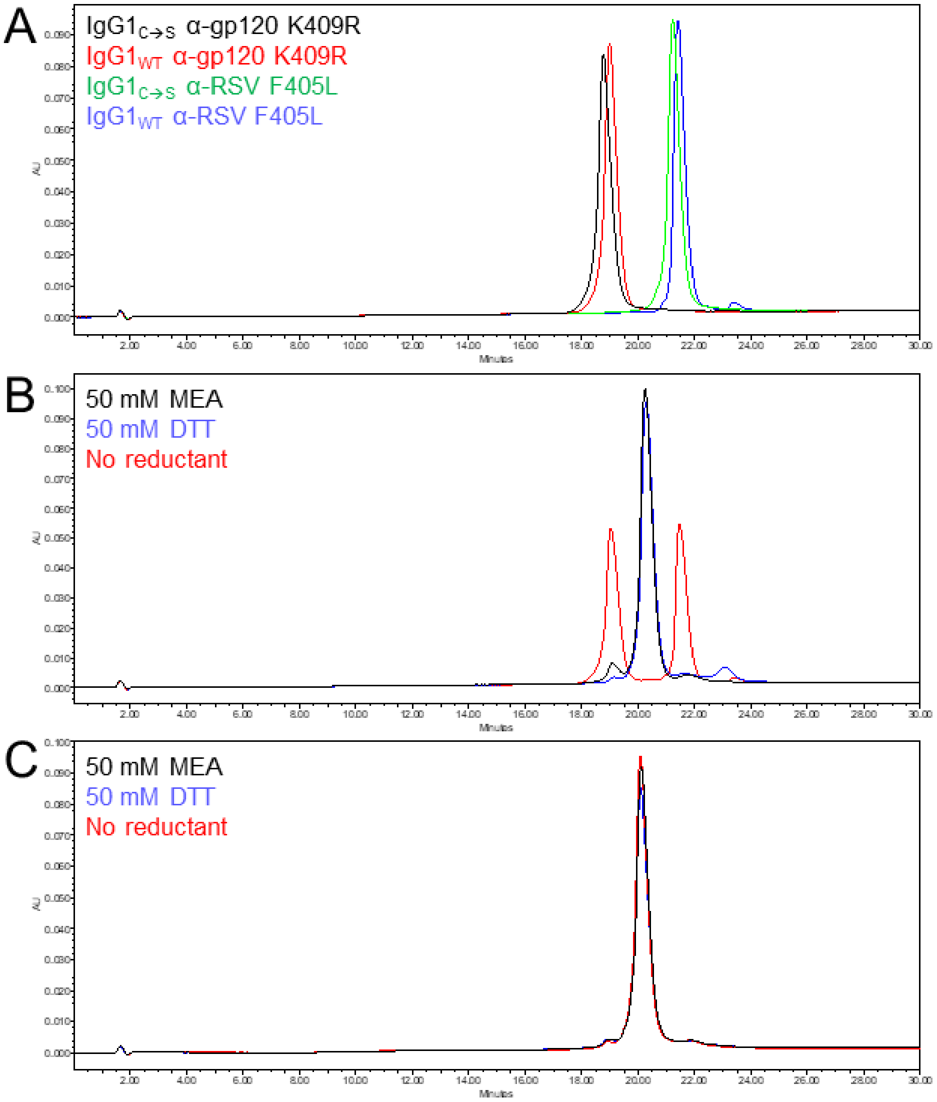

Figure S3. Hydrophobic interaction was used to separate parental Abs, with IgG1$_{WT}$ proteins at similar retention times as the corresponding IgG1$_{C\rightarrow S}$ proteins (A). When cFAE was performed with IgG1$_{WT}$ proteins in the presence of MEA or DTT, a peak corresponding to bsAb appeared at a retention time between those of parental Abs, but no bsAb was formed in the absence of reducing agent (B). When cFAE was performed with IgG1$_{C\rightarrow S}$ proteins, full formation of bsAb was observed with MEA or DTT, and in the absence of reducing agent (C). Absorbance at 280 nm is plotted versus retention time in minutes.

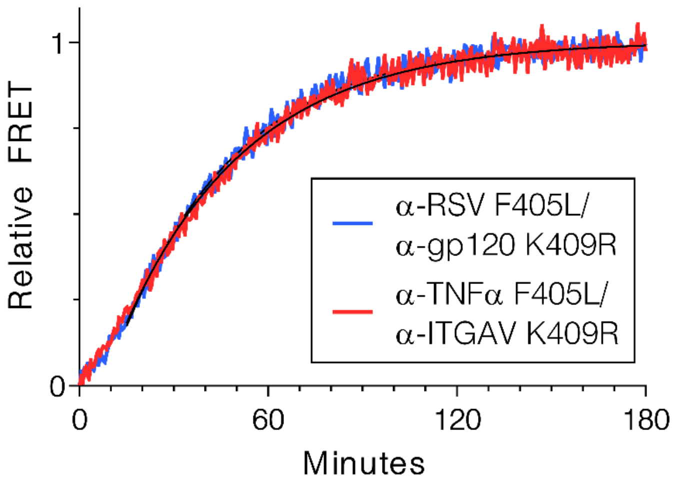

Figure S4. Kinetics of cFAE based on an increase in FRET for two pairs of IgG1 Abs targeting different antigens. Blue trace shows the cFAE reaction for Abs targeting respiratory syncytial virus (RSV) and HIV glycoprotein gp120, while red trace shows the same reaction for Abs targeting tumor necrosis factor α (TNFα) and integrin alpha-V (ITGAV). Both experiments contained 50 mM of 2-mercaptoethylamine and 100 nM of each Ab at 25 ºC, and traces show the average of both Ab-dye pairings (F405L Ab labeled with Alexa 488 and K409R Ab labeled with Alexa 594, or the opposite pairing of Ab and dye). The rate for α-RSV/α-gp120 (omitting the first 15 minutes containing reduction-related lag phase) was 0.0268 ± 0.0008 min^{-1} and for α-TNFα/α-gp120 was 0.0251 ± 0.0011 min^{-1}.

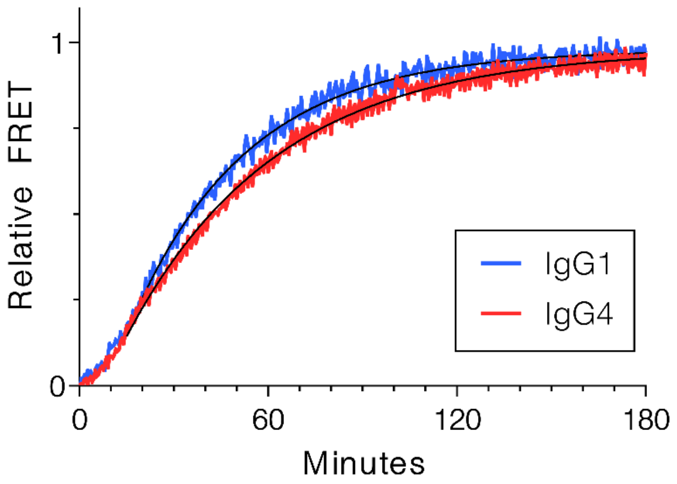

Figure S5. Kinetics of cFAE based on an increase in FRET for two sets of Abs having the same variable regions, but the framework of IgG1 or IgG4PAA (S228P/L234A/L235A). IgG1 Abs undergoing cFAE were α-RSV F405L and α-gp120 K409R, while IgG4 Abs were α-RSV F405L/R409K and α-gp120 (naturally having R409). Reactions contained 50 mM of 2-mercaptoethylamine and 100 nM of each Ab at 25 ºC, and traces show the average of both Ab-dye pairings. The rate (omitting the first 15 minutes containing reduction-related lag phase) for IgG1 was 0.0268 ± 0.0008 min^{-1} and for IgG4 was 0.0209 ± 0.0019 min^{-1}.

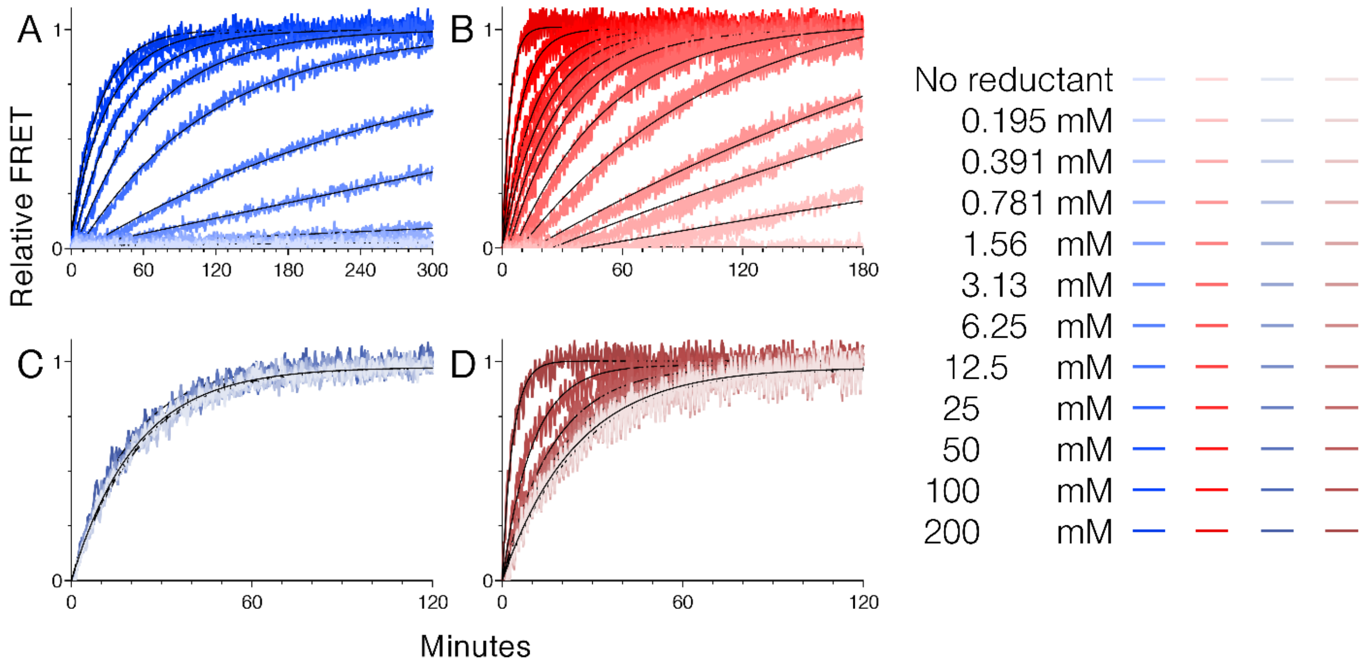

Figure S6. Kinetics of cFAE based on an increase in FRET for the formation of bispecific $IgG1_{WT}$ (A, B) or $IgG1_{C\rightarrow S}$ (C, D) in the presence of varying concentrations of MEA (A, C) or DTT (B, D) at 25 ºC. Concentration of reducing agent is denoted by the luminance of the trace, with the highest concentration of 200 mM as the darkest color, 1:2 dilutions in progressively lighter colors, and no reductant as the lightest colored trace. Rates from monoexponential fits are plotted in Figure 2 of the main text.

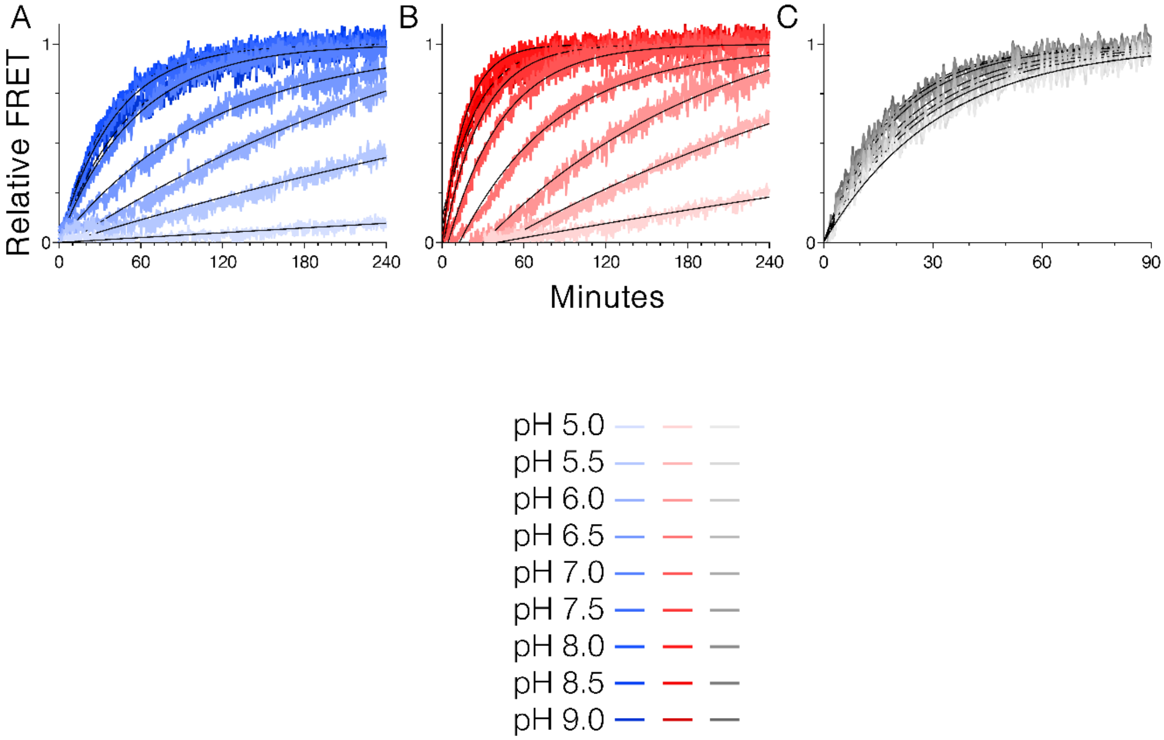

Figure S7. Kinetics of cFAE based on an increase in FRET for the formation of bispecific $IgG1_{WT}$ with 50 mM MEA (A), $IgG1_{WT}$ with 10 mM DTT (B), or $IgG1_{C\rightarrow S}$ (C) at varying pH. The cFAE reaction was performed in 100 mM potassium phosphate, 100 mM sodium chloride at pH 5 to 9 and at 25 ºC. As the pH is increased, kinetic traces are colored progressively darker. Rates from monoexponential fits are plotted in Figure 3A of the main text.

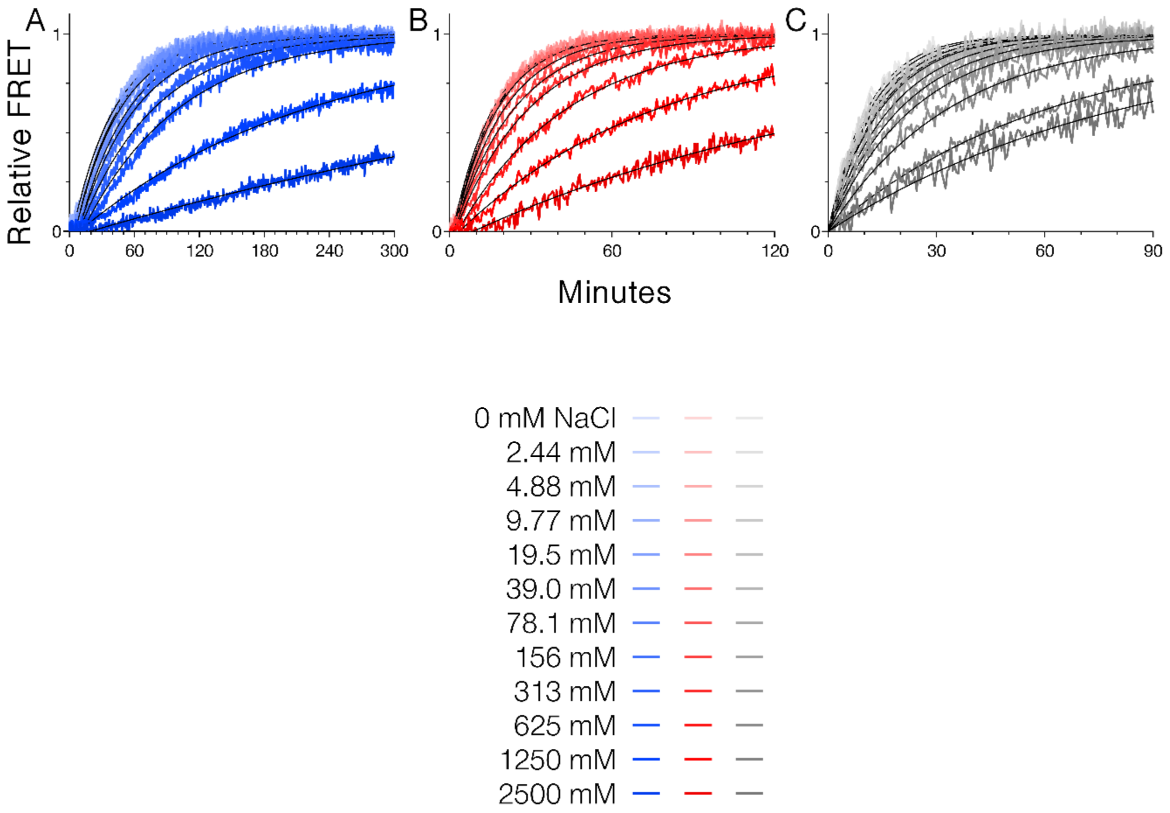

Figure S8. Kinetic FRET data showing progress of cFAE over time for the formation of bispecific $IgG1_{WT}$ with 50 mM MEA (A), $IgG1_{WT}$ with 10 mM DTT (B), or $IgG1_{C \to S}$ (C) at varying ionic strength. The cFAE reaction was performed in 20 mM potassium phosphate, pH 7.4 containing 0 to 2.5 M sodium chloride at 25 ºC. As the NaCl concentration is increased, kinetic traces are colored progressively darker. Rates from monoexponential fits are plotted in Figure 3B of the main text.

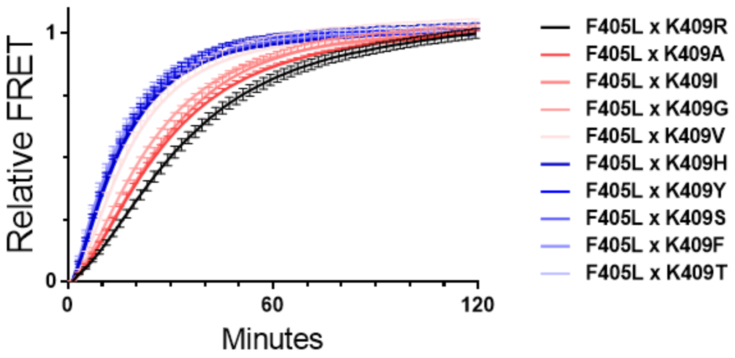

Figure S9. Kinetic FRET data showing progress of cFAE over time for the formation of bispecific IgG1-WT using a F405L parental Ab paired with parental Abs containing different K409 substitutions. The cFAE reaction was performed in PBS, pH 7.3 at 25 ºC and contained 25 mM 2-MEA and 167 nM of total Ab. The rate for the F405L x K409R pair was 0.027 ± 0.001 min^{-1}. The alternative K409 substitutions increased the rates ranging from 0.031 ± 0.001 min^{-1} (F405L x K409A) to 0.050 ± 0.002 min^{-1} (F405L x K409S).

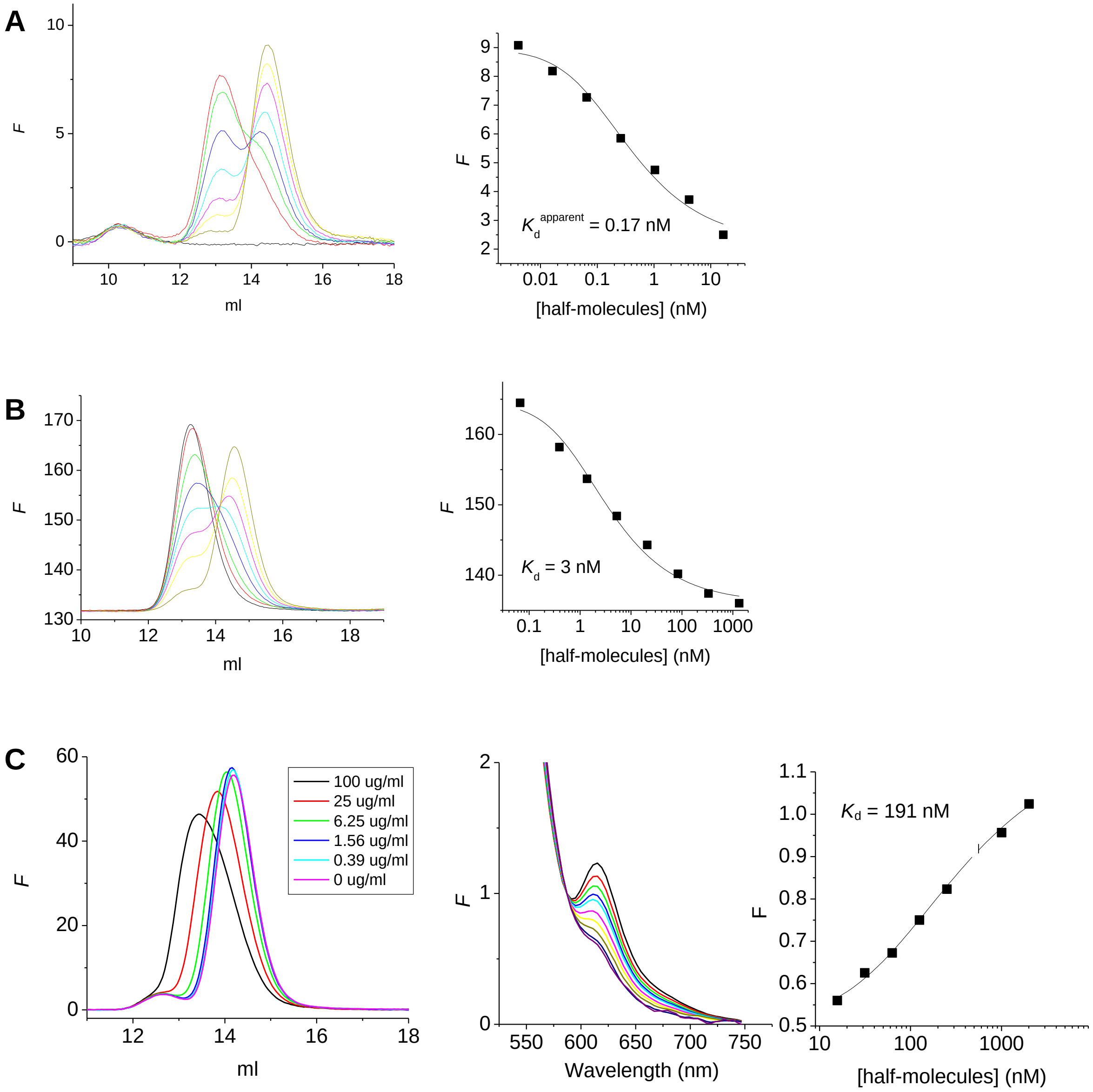

Figure S10. Analysis of bsAb interaction strength. A fixed amount of DyLight488-labeled F405L (A and C) or DyLight488-labeled K409R (B) was incubated with different concentrations of unlabeled bsAb (A), K409R (B) or F405L (C) parental (all reduced), respectively, and analyzed with high performance size-exclusion chromatography (left panels). Fluorescence at 14.5 ml is plotted vs concentration of half-molecules (right panels) and analyzed to calculate the K_D value.
The F405L dissociated rapidly and with the column assay a peak shift was observed rather than two populations (half-molecules and homo-dimers). The dissociation constant was therefore alternatively measured using a FRET assay (C; middle and right panels): Equimolar amounts of DyLight488 and DyLight594-labeled F405L were incubated at various concentrations in the presence of DTT and fluorescence spectra recorded. Normalized fluorescence is plotted vs concentration of half-molecules and analyzed to calculate the K_D value.

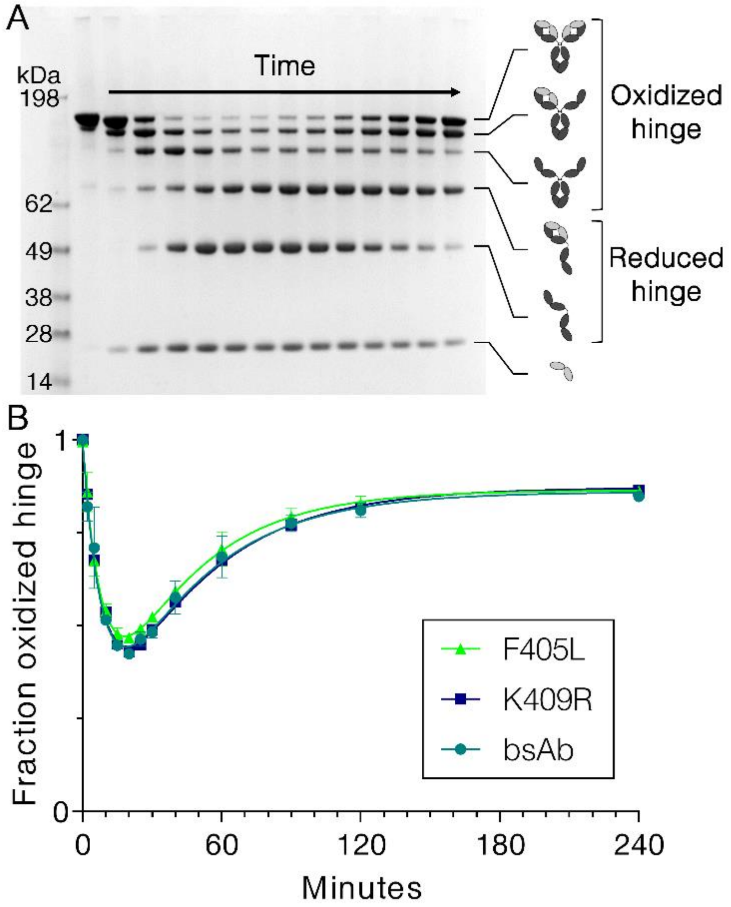

Figure S11. Kinetics of hinge reduction and re-oxidation by non-reducing SDS-PAGE, analogous to Figure 5 in the main text. Two mg/mL of α-RSV F405L, 2 mg/mL of α-gp120 K409R, or 1 mg/mL of each were reduced with 50 mM MEA. The reaction was incubated at 300 rpm and 31 ºC, and time points were quenched by adding an excess of iodoacetamide. After performing SDS-PAGE densitometry to determine the fraction of oxidized hinge disulfides at each point and fitting the kinetics to the sum of two exponentials, rates of reduction were 0.0890 ± 0.0022 min^{-1} for F405L, 0.0894 ± 0.0041 min^{-1} for K409R, and 0.0924 ± 0.0038 min^{-1} for the mixture; and rates of oxidation were 0.0276 ± 0.0009 min^{-1} for F405L, 0.0240 ± 0.0016 min^{-1} for K409R, and 0.0252 ± 0.0015 for the mixture.

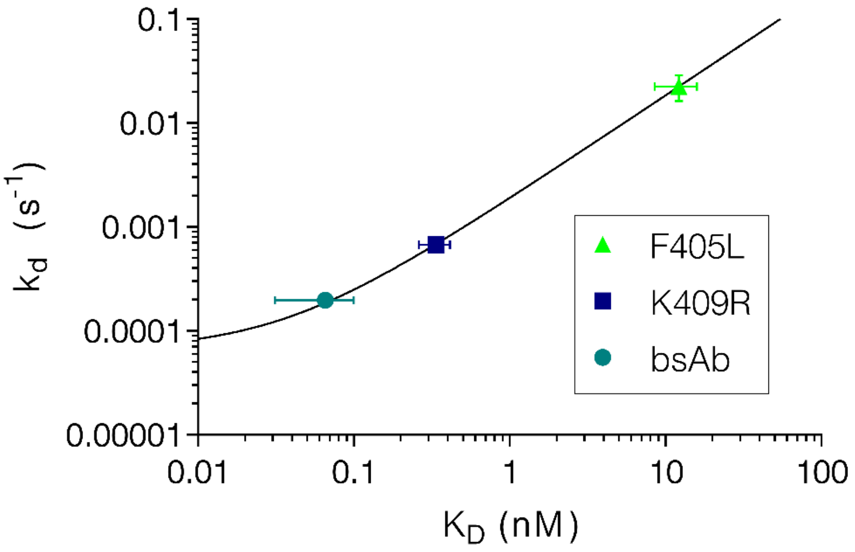

Figure S12. Comparison of half-Ab dissociation rates and half-Ab dimerization affinities. Y-axis is the half-Ab dissociation rate, measured by FRET with different pairs of Alexa 488- and Alexa 594-labeled $IgG1_{C\rightarrow S}$. X-axis is the half-Ab dimerization affinity, measured by FCS with Alexa 488-labeled half-Fc binding to unlabeled half-Ab. Values of k_d and K_D were tightly correlated with $R^2 = 1$. Note that the linear fit appears curved due to the log axes.

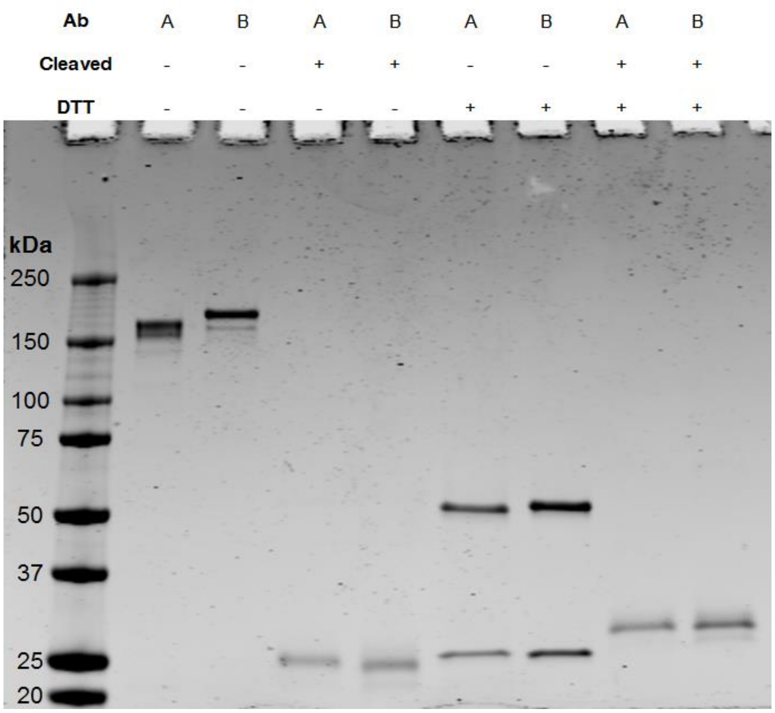

Figure S13. SDS-PAGE of parental Abs and purified Fc fragments. Ab A is α-RSV F405L and Ab B is α-gp120 K409R, both in IgG1. Samples were labeled with 10x Alexa 488 NHS ester and subsequently treated with IdeS enzyme (FabRICATOR, Genovis) per the manufacturer's instructions. After the reactions, Fc was purified by protein A chromatography and polished by SEC to remove uncleaved Ab. Experiment was performed by running a Mini-PROTEAN TGX 4-15% gel (Bio-Rad, 4561083) at 150 V using samples with or without 50 mM DTT.

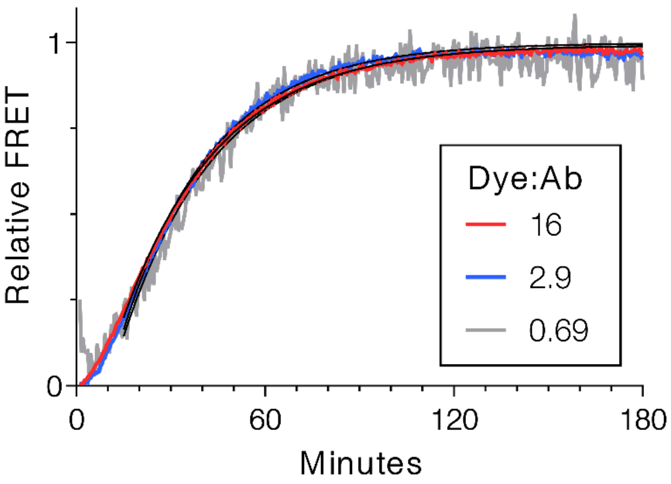

Figure S14. Kinetics of cFAE based on an increase in FRET for Abs containing different extents of labeling. Abs were labeled with a low, intermediate, or high concentration of dye to achieve varying ratios of dye:Ab and then cFAE was performed with IgG1 α-RSV F405L and α-gp120 K409R. Reactions contained 50 mM of 2-mercaptoethylamine and 500 nM of each Ab and were performed in duplicate at 25 ºC. The rate for the lowest labeled pair was 0.0253 ± 0.0001 min^{-1}, for the moderately labeled pair was 0.0294 ± 0.0026 min^{-1}, and for the heavily labeled pair was 0.0281 ± 0.0004 min^{-1}.

Scheme S1. Derivation of the equilibrium constant for parental Abs and bsAb

Consider the following half-Ab homodimerization equilibria and corresponding K_D definitions, with half-Ab A containing F405L and half-Ab B containing K409R. AA and BB then represent full parental Abs containing two half-Abs with the respective mutations. Note that these equilibria are true only in the absence of hinge disulfide bonds (as occurs under reducing conditions and with $IgG1_{C \rightarrow S}$ or IgG1 Fc cleaved below the hinge).

$$AA \overset{K_{AA}}{\longleftrightarrow} 2A \qquad K_{AA} = \frac{[A]^2}{[AA]}$$

$$BB \overset{K_{BB}}{\longleftrightarrow} 2B \qquad K_{BB} = \frac{[B]^2}{[BB]}$$

In addition, A and B can heterodimerize to form bsAb AB.

$$AB \overset{K_{AB}}{\longleftrightarrow} A + B \qquad K_{AB} = \frac{[A][B]}{[AB]}$$

The cFAE reaction to form bsAb from two parental Abs containing cFAE mutations can then be considered in terms of the following net equilibrium, where the value of K_{eq} represents the preference for bsAb compared to parental Abs.

$$AA + BB \overset{K_{eq}}{\longleftrightarrow} 2AB \qquad K_{eq} = \frac{[AB]^2}{[AA][BB]}$$

To obtain K_{eq} in terms of individual K_D values, [AB], [AA], and [BB] can be substituted based on the individual K_D definitions.

$$K_{eq} = \frac{[AB]^2}{[AA][BB]} = \frac{\left(\frac{[A][B]}{K_{AB}}\right)^2}{\left(\frac{[A]^2}{K_{AA}}\right)\left(\frac{[B]^2}{K_{BB}}\right)} = \frac{K_{AA}K_{BB}}{K_{AB}^2}$$

Scheme S2. Calculation of %bsAb from K_{eq}

When cFAE is performed, AA are BB are incubated at equimolar concentrations in the presence of reducing agent, which allows AA, BB, and AB to reach equilibrium. Thus, [AA]=[BB] at the start of the reaction, and assuming concentrations are high enough to preclude significant [A] and [B] this is also true at equilibrium. This condition allows [AA], [BB], and [AB] to be rewritten in terms of [parental Ab] and [bsAb].

$$[AA] = [BB] = \frac{[Parental\ Ab]}{2}$$

$$[AB] = [bsAb]$$

These new definitions can be used in the previously derived equation for K_{eq}.

$$K_{eq} = \frac{[AB]^2}{[AA][BB]} = \frac{[bsAb]^2}{\left(\frac{[Parental\ Ab]}{2}\right)^2} = \frac{4[bsAb]^2}{[Parental\ Ab]^2}$$

Solving for [Parental Ab] yields

$$[Parental\ Ab] = \frac{2[bsAb]}{\sqrt{K_{eq}}}$$

Finally, the fraction of bsAb can be written in terms of [bsAb] and [Parental Ab].

$$Fraction\ bsAb = \frac{[bsAb]}{[bsAb] + [Parental\ Ab]}$$

Substituting for [Parental Ab] allows for expression of "fraction bsAb" in terms of K_{eq}.

$$Fraction\ bsAb = \frac{[bsAb]}{[bsAb] + \frac{2[bsAb]}{\sqrt{K_{eq}}}} = \frac{1}{1 + \frac{2}{\sqrt{K_{eq}}}}$$

Scheme S3. Derivation of FCS fitting equations

The K_D for the heterodimerization of half-Fc A and half-Ab B depends on the relative amounts of free half-Fc, A_f, and free half-Ab, B_f, compared to the amount of the complex at equilibrium.

$$AB \overset{K_{AB}}{\longleftrightarrow} A_f + B_f \qquad K_{AB} = \frac{[A_f][B_f]}{[AB]}$$

However, A_f and B_f can also homodimerize to the corresponding full Fc, AA, and full Ab, BB.

$$AA \overset{K_{AA}}{\longleftrightarrow} 2A_f \qquad K_{AA} = \frac{[A_f]^2}{[AA]}$$

$$BB \overset{K_{BB}}{\longleftrightarrow} 2B_f \qquad K_{BB} = \frac{[B_f]^2}{[BB]}$$

The total half-Fc concentration A_t can be expressed in terms of the free half-Fc concentration, the concentration of the dimerized full Fc, and the concentration of the half-Fc/half-Ab heterodimer. The corresponding equation is true for B_t.

$$[A_t] = [A_f] + 2[AA] + [AB]$$

$$[B_t] = [B_f] + 2[BB] + [AB]$$

With five equations and five dependent variables (A_f, B_f, AA, BB, AB), it is then possible to solve for any dependent variable in terms of independent variables (A_T, B_T, K_{AA}, K_{BB}, K_{AB}). Thus, explicit equations were determined for A_f, AA, and AB using the Solve function of Mathematica 11.1. Since there were four solutions to each quartic function, ranges of values for K_{AA}, K_{BB}, and K_{AB} were tested for each solution to determine its viable domain. Solution 1 was valid when K_{AB} was less than both K_{AA} and K_{BB} while equation 3 was valid when K_{AB} was greater than or equal to either K_{AA} or K_{BB}. Thus, equation 1 was used for heterodimerization reactions while equation 3 was used for homodimerization reactions (when K_{AA}=K_{BB}=K_{AB}).

The observed diffusion time is equal to the diffusion time of each species multiplied by the relative amount of that species. Thus, the diffusion times observed during titrations can be modeled using the solved equations for A_f, AA, and AB.

$$\tau_{D,obs} = \frac{[A_f]}{[A_t]}\tau_{D,Af} + \frac{2[AA]}{[A_t]}\tau_{D,AA} + \frac{[AB]}{[A_t]}\tau_{D,AB}$$

Since individual species A_f, AA, and AB cannot be isolated, it is difficult to determine their precise diffusion times. However, the general relation between mass and diffusion time should hold such that

$$\frac{\tau_{D,bound}}{\tau_{D,unbound}} = \sqrt[3]{\frac{MW_{bound}}{MW_{unbound}}}$$

Therefore, the increase in diffusion time for AA and AB relative to A_f can be calculated based on the mass of each species. As a result, the observed diffusion time can be modeled to the equation below.

$$\tau_{D,obs} = \frac{[A_f]}{[A_t]}\tau_{D,Af} + \frac{2.52[AA]}{[A_t]}\tau_{D,Af} + \frac{1.59[AB]}{[A_t]}\tau_{D,Af}$$

As $[A_f]$, [AA], [AB] are equations in terms of A_T, B_T, K_{AA}, K_{BB}, and K_{AB}, the $\tau_{D,obs}$ equation was fit by fixing known values of A_T and B_T while letting K_{AA}, K_{BB}, K_{AB}, and $\tau_{D,Af}$ float. By fitting globally across datasets for each combination of half-Fc and half-Ab, it was possible to determine K_D values for F405L homodimerization, K409R homodimerization, and bsAb heterodimerization with higher confidence. The relevant K_Ds for each interaction are tabulated below. It was assumed that the affinity of a given half-Fc homodimer, half-Ab homodimer, and half-Fc/half-Ab heterodimer were equal since the interaction occurs predominantly in the C_H3 domain.

**Half-Fc**	**Half-Ab**	**K_{AA}**	**K_{BB}**	**K_{AB}**
F405L	F405L	F405L	F405L	F405L
F405L	K409R	F405L	K409R	bsAb
K409R	F405L	K409R	F405L	bsAb
K409R	K409R	K409R	K409R	K409R

Supplementary References

1. Teeling, J. L., French, R. R., Cragg, M. S., Van den Brakel, J., Pluyter, M., Huang, H., Chan, C., Parren, P. W. H. I., Hack, C. E., Dechant, M., Valerius, T., Van de Winkel, J. G. J., and Glennie, M. J. (2004) Characterization of new human CD20 monoclonal antibodies with potent cytolytic activity against non-Hodgkin lymphomas. *Blood*. **104**, 1793–1800
2. Bleeker, W. K., van Bueren, J. J., van Ojik, H. H., Gerritsen, A. F., Pluyter, M., Houtkamp, M., Halk, E., Goldstein, J., Schuurman, J., van Dijk, M. A., van de Winkel, J. G. J., and Parren, P. W. H. I. (2004) Dual mode of action of a human anti-epidermal growth factor receptor monoclonal antibody for cancer therapy. *J. Immunol.* **173**, 4699–4707
3. Gramer, M. J., van den Bremer, E. T. J., van Kampen, M. D., Kundu, A., Kopfmann, P., Etter, E., Stinehelfer, D., Long, J., Lannom, T., Noordergraaf, E. H., Gerritsen, J., Labrijn, A. F., Schuurman, J., Van Berkel, P. H. C., and Parren, P. W. H. I. (2013) Production of stable bispecific IgG1 by controlled Fab-arm exchange: scalability from bench to large-scale manufacturing by application of standard approaches. *MAbs*. **5**, 962–973
4. Labrijn, A. F., Meesters, J. I., de Goeij, B. E. C. G., van den Bremer, E. T. J., Neijssen, J., van Kampen, M. D., Strumane, K., Verploegen, S., Kundu, A., Gramer, M. J., van Berkel, P. H. C., van de Winkel, J. G. J., Schuurman, J., and Parren, P. W. H. I. (2013) Efficient generation of stable bispecific IgG1 by controlled Fab-arm exchange. *Proc. Natl. Acad. Sci. U. S. A.* **110**, 5145–5150
5. Zhou, H., Jang, H., Fleischmann, R. M., Bouman-Thio, E., Xu, Z., Marini, J. C., Pendley, C., Jiao, Q., Shankar, G., Marciniak, S. J., Cohen, S. B., Rahman, M. U., Baker, D., Mascelli, M. A., Davis, H. M., and Everitt, D. E. (2007) Pharmacokinetics and safety of golimumab, a fully human anti-TNF-alpha monoclonal antibody, in subjects with rheumatoid arthritis. *J. Clin. Pharmacol.* **47**, 383–396
6. Trikha, M., Zhou, Z., Nemeth, J. A., Chen, Q., Sharp, C., Emmell, E., Giles-Komar, J., and Nakada, M. T. (2004) CNTO 95, a fully human monoclonal antibody that inhibits αv integrins, has antitumor and antiangiogenic activity in vivo. *Int. J. Cancer*. **110**, 326–335
